# Design and validation of a DNA-microarray for phylogenetic analysis of bacterial communities in different oral samples and dental implants

**DOI:** 10.1038/s41598-017-06743-6

**Published:** 2017-07-24

**Authors:** Carola Parolin, Barbara Giordani, Rogers Alberto Ñahui Palomino, Elena Biagi, Marco Severgnini, Clarissa Consolandi, Giada Caredda, Stefano Storelli, Laura Strohmenger, Beatrice Vitali

**Affiliations:** 1 Department of Pharmacy and Biotechnology, University of Bologna, Bologna, Italy; 2 Institute of Biomedical Technologies – National Research Council, Segrate, Milan Italy; 3 Dental Clinic, Department of Biomedical, Surgical and Dental Sciences, ASST Santi Paolo e Carlo, University of Milan, Milan, Italy

## Abstract

The quali-quantitative characterization of the oral microbiota is crucial for an exhaustive knowledge of the oral ecology and the modifications of the microbial composition that occur during periodontal pathologies. In this study, we designed and validated a new phylogenetic DNA-microarray (OralArray) to quickly and reliably characterize the most representative bacterial groups that colonize the oral cavity. The OralArray is based on the Ligation Detection Reaction technology associated to Universal Arrays (LDR-UA), and includes 22 probe sets targeted to bacteria belonging to the phyla *Firmicutes*, *Proteobacteria*, *Actinobacteria*, *Bacteroidetes*, *Fusobacteria*, and *Spirochaete*. The tool is characterized by high specificity, sensitivity and reproducibility. The OralArray was successfully tested and validated on different oral samples (saliva, lingual plaque, supragingival plaque, and healing cap) collected from 10 healthy subjects. For each specimen, a microbial signature was obtained, and our results established the presence of an oral microbial profile specific for each subject. Moreover, the tool was applied to evaluate the efficacy of a disinfectant treatment on the healing caps before their usage. The OralArray is, thus, suitable to study the microbiota associated with various oral sites and to monitor changes arising from therapeutic treatments.

## Introduction

Understanding the role of the microbial communities associated to the human body is emerging as one of the most important and fascinating biomedical challenges of our times^1–3^. The commensal human microbiome has recently been estimated to equal the amount of the human body cells^4^. Complex microbial communities are normal residents of the skin, oral cavity, vaginal, and intestinal mucosa and carry a broad range of functions essential for the wellbeing of the host^5^. When the balance of these communities is altered, opportunistic or pathogenic species can take over, causing, in some cases, infection and inflammation. In contrast to the traditional view of individual pathogens being responsible for disease onset, recent phylogenetic studies seem to point to a new perspective in which the transition from health to disease is attributed to a shift in the global balance of the microbiota rather than to the specific appearance of individual pathogens^3, 6^.

In recent years, next-generation massive sequencing (NGS) has allowed the complete sequencing of an ever increasing number of commensals and opportunistic bacteria related to human niches, such as gut, skin, vagina, and mouth. At the same time, NGS has been, also, applied to the description of microbial communities, increasing our knowledge of the microbiome inhabitants^7^.

Efforts to characterize microbial diversity increasingly, at present, rely on cultivation-independent, molecular techniques^8, 9^, since the vast majority of bacteria are not cultivable. Most of these molecular studies are based on the small subunit (16 S) of ribosomal RNA (rRNA) gene because of its universal presence in cellular organisms, the presence of conserved regions, and its reliability for phylogenetic analysis^10^.

Using rRNA gene-based techniques, it is estimated that the human oral cavity harbors ca. 700 different bacterial species^11–13^, that reach the number of 10^11^ CFU/g in dental plaque and 10^8^–10^9^ CFU/g in saliva^14, 15^. Some of these bacteria are closely related to the development of oral diseases, mainly dental caries and periodontitis^9, 16^. The oral microbiota also seems to be involved in several non-oral diseases, such as bacterial endocarditis, heart disease, obesity, pneumonia, atherosclerosis, and preterm low birth weight^17–22^.

Phylogenetic DNA-microarrays have been recognized in the scientific community as valuable tools for a high-throughput, quantitative and systematic analysis of bacterial communities in different human microbial ecosystems^23–26^, including the oral microbiota^24^. The objective of the present study was to develop a new phylogenetic DNA-microarray, named OralArray, to quickly and reliably characterize a selected core of representative bacterial groups that colonize different sites of the oral cavity. The peculiarity of the OralArray is the association of the Ligation Detection Reaction technology with Universal Arrays (LDR-UA)^27–29^. This technique relies on the ligation of two probe types (one labeled Discriminating Probe, DP; and one Common Probe, CP, forming together one probe set), operated by a ligase upon perfect match with the target molecule. The ligation event is visualized on a UA carrying Zip Codes, a set of artificial sequences, that are complementary to the cZip Codes fused to CPs.

The OralArray was successfully tested and validated on different oral samples, saliva, lingual plaque, supragingival plaque and healing cap (control and pretreated with chlorhexidine), demonstrating its potential to study the microbiota associated with various oral sites and to monitor changes arising from therapeutic treatments.

## Results

### Probe panel of the OralArray

**Figure 1:**
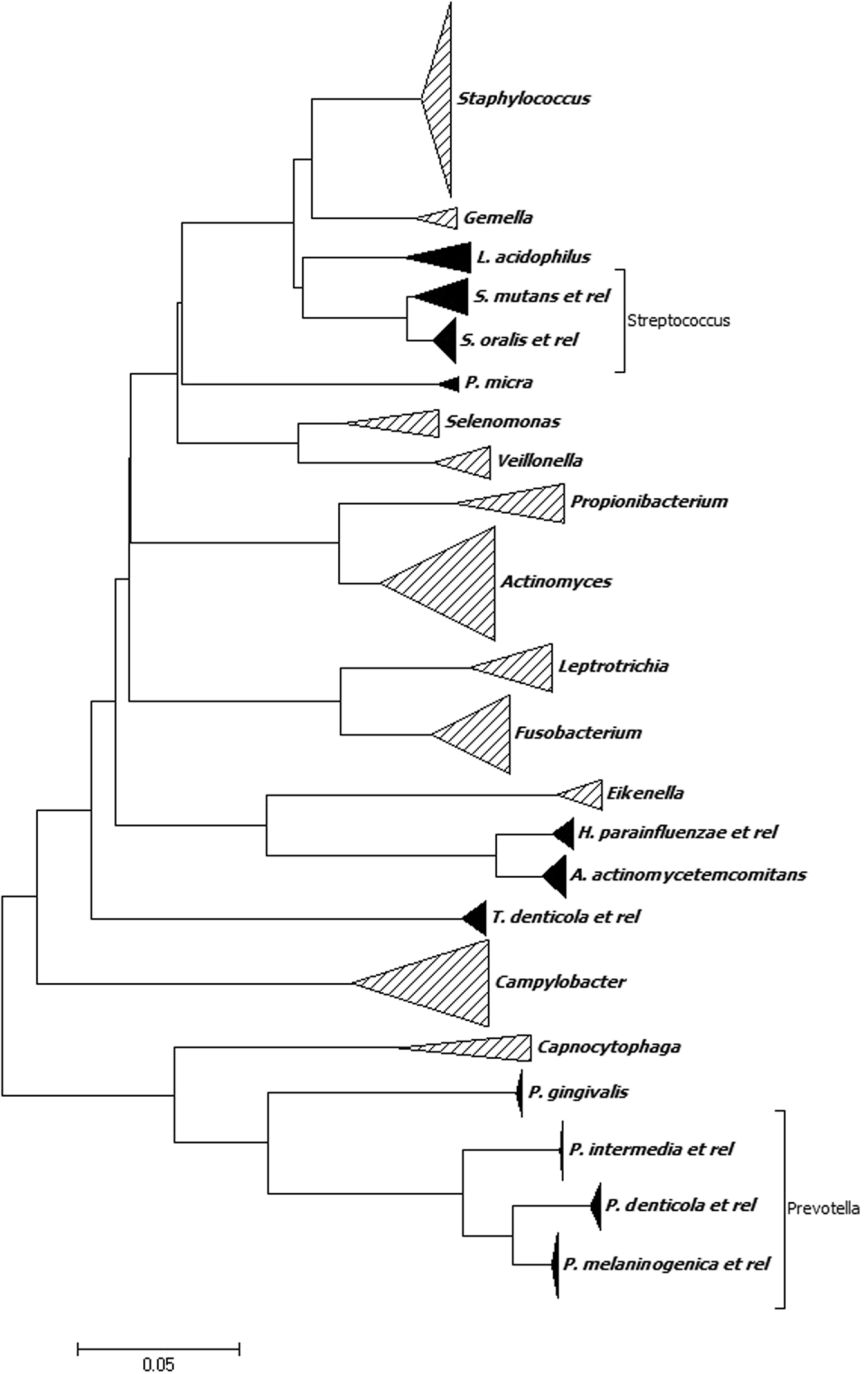
Phylogenetic tree of the oral microrganisms targeted by the OralArray. The tree was obtained from the entire positive set used for the probe design. The neighbor-joining method was used to infer evolutionary history. The evolutionary distances were computed using the maximum composite likelihood method and units correspond to the number of base substitutions per site. The analysis involved 383 nucleotide sequences (Supplementary Table S2). All positions containing gaps and missing data were eliminated. Evolutionary analyses were conducted by using MEGA6 software.

Based on the literature available in the field of human oral ecosystem and the most relevant oral pathologies^3, 5, 30–34^, twenty two bacterial targets were selected (Table 1) and specific probe sets were designed by using ORMA probe designer tool^35^, as described in Materials and Methods section. A phylogenetic tree was built by using all positive sets (Fig. 1). The targets belong to the prokaryotic phyla *Firmicutes*, *Proteobacteria*, *Actinobacteria*, *Bacteroidetes, Fusobacteria*, and *Spirochaetes*, which account for 96% of the species detected in the oral microbiome^5, 32^. The probes were designed to cover different phylogenetic levels, from a single representative species, to entire genera. Within *Firmicutes*, the genus *Streptococcus* is the most representative in the mouth: a probe set targeting *S. oralis* and its related species (i.e. *S. mitis*, *S. infantis, S. pneumoniae*) and one set targeting the cariogenic species *S. mutans* were designed. The other known cariogenic species *Lactobacillus acidophilus* was addressed by a specific probe set previously designed and validated on a LDR-UA platform for the characterization of the vaginal microbiota (VaginArray^26^). The *Firmicutes* genera *Gemella* and *Staphylococcus* are, also, extremely common in the oral ecosystem, thus they were targeted by using genus-level probe sets; *Staphylococcus* probe set was mutuated from the VaginArray^26^. Within the *Clostridia* class of *Firmicutes*, the species *Parvimonas micra* was selected as recurrent in the periodontal disease^36^ and a probe pair was designed; the genera *Selenomonas* and *Veillonella* are the most representative of the *Veillonellaceae* family^37^ and were targeted by two genus-level probe sets. The phylum *Fusobacteria* includes the genera *Fusobacterium* and *Leptotrichia*, frequently detected in oral samples, as well as in other human ecosystem, and were recognized by two genus-level probe sets, both already validated in previous LDR-UA-based assays (HTF-MicroBi.Array^25^ and VaginArray^26^, respectively). Within the phylum *Actinobacteria*, *Actinomyces* and *Propionibacterium* genera have been associated to gingivitis and dental caries^38^ and have been addressed by two genus-level probe sets. All human oral taxa identified to date in the phylum *Spirochaetes* are members of the genus *Treponema* ^37^, in particular the most recurrent species *T. denticola* and *T. putidum*, thus a group-specific probe pair was designed. For the phylum *Proteobacteria*, two genus-specific probe sets were included; one, specifically designed to detect the genus *Eikenella*, the other, targeting the genus *Campylobacter*, has been already reported in Candela *et al*.^25^. In addition, a probe set addressing the peri implantitis-related species *Aggregatibacter actinomycetemcomitans* and a probe set for the healthy marker *Haemophilus parainfluenzae* and the related species *H. ducreyi* and *H. pittmaniae* were developed. Among *Bacterioidetes* phylum, *Prevotella* is the largest genus and was addressed by three group-specific probe sets: the first recognized *P. denticola* and the related species *P. multiformis*, the second recognized *P. intermedia, P. falsenii*, and *P. nigrescens*, the last detected *P. melaninogenica* and the related species *P. veroralis* and *P. histicola*. For the *Bacterioidetes* phylum, the genus *Capnocytophaga* and the periodontitis-associated species *Porphyromonas gingivalis* were selected and targeted in the array, too.

**Table 1:** Bacterial targets of the OralArray, the taxonomic level, the phylogenetic classification and the Zip Code associated in the array.

Probe set	Taxonomic level	Phylum	Zip Code
*Streptococcus oralis* and rel.	Cluster	Firmicutes	3
*Streptococcus mutans*	Species	Firmicutes	1
*Lactobacillus acidophilus*	Species	Firmicutes	23
*Gemella*	Genus	Firmicutes	5
*Staphylococcus*	Genus	Firmicutes	29
*Parvimonas micra*	Species	Firmicutes	9
*Selenomonas*	Genus	Firmicutes	7
*Veillonella*	Genus	Firmicutes	15B
*Leptotrichia*	Genus	Fusobacteria	8
*Fusobacterium*	Genus	Fusobacteria	15
*Propionibacterium*	Genus	Actinobacteria	17
*Actinomyces*	Genus	Actinobacteria	12
*Treponema denticola* and rel.	Cluster	Spirochaetes	1B
*Eikenella*	Genus	Proteobacteria	21
*Haemophilus parainfluenzae* and rel.	Cluster	Proteobacteria	27
*Aggregatibacter actinomycetemcomitans*	Species	Proteobacteria	31
*Campylobacter*	Genus	Proteobacteria	6
*Capnocytophaga*	Genus	Bacteroidetes	41
*Porphyromonas gingivalis*	Species	Bacteroidetes	39
*Prevotella denticola* and rel.	Cluster	Bacteroidetes	33
*Prevotella intermedia* and rel.	Cluster	Bacteroidetes	35
*Prevotella melaninogenica* and rel.	Cluster	Bacteroidetes	37

The newly designed probes had an average melting temperature (Tm) of 67.7 ± 0.3 °C (n = 34) and an average length of 36 ± 5.3 nucleotides (Supplementary Table S1). Seven probes out of thirty-four contained degenerated bases, in particular four probes contained only one ambiguous base, one probe contained 2 ambiguous bases and two probes contained 3 ambiguous bases.

### Validation of the OralArray on DNA samples from pure bacterial cultures: specificity, reproducibility and sensitivity

The specificity of the designed probe sets was tested by using 10 ng of 16 S rRNA PCR products from twenty-two microorganisms, members of the human oral microbiota (Table 2). Moreover, the probe sets designed for HTF-MicroBi.Array^25^ and the VaginArray^26^ (already validated towards gastrointestinal and vaginal microorganisms, respectively) were subjected to specificity tests by using oral microbial species. Amplicons were prepared by PCR of genomic DNA provided by DSMZ or extracted from pure cultures. All 16 S rRNA amplicons were properly recognized in separate LDR hybridization reactions with the entire probe panel of the array: only probes associated with the expected species and control spots were significantly over the background, with P-values < 0.0005. Signal-to-noise ratios (SNR) calculated for the spots called as significant (sSNR) varied from 12.94 to 279.57, with an average of 64.96, whereas SNR of the remaining spots (not significant spots, nsSNR) varied from 0.89 to 1.71, with an average of 1.36 (Table 2).

**Table 2:** Bacterial species used for specificity tests, P-values, probe specific (sSNR) and not specific (nsSNR) signal-to-noise ratios.

Probe set	Species	P-value	sSNR	nsSNR
*S. oralis* and rel.	*S. oralis*	7.20 × 10^–9^	168.65	1.61
*S. mutans*	*S. mutans*	2.49 × 10^-6^	60.06	1.61
*L. acidophilus*	*L. acidophilus*	9.24 × 10^−7^	51.45	1.56
*Gemella*	*G. morbillorum*	2.04 × 10^−4^	100.48	1.52
*Staphylococcus*	*S. aureus*	1.81 × 10^−4^	55.16	1.23
*P. micra*	*P. micra*	2.85 × 10^−6^	16.77	1.38
*Selenomonas*	*S. noxia*	1.17 × 10^−6^	100.34	1.71
*Veillonella*	*V. parvula*	4.27 × 10^−6^	127.11	1.45
*Leptotrichia*	*L. buccalis*	1.76 × 10^−8^	12.94	0.89
*Fusobacterium*	*F. nucleatum subsp. polymorphum*	5.56 × 10^−11^	279.57	1.67
*Propionibacterium*	*P. acidifaciens*	3.34 × 10^−12^	143.65	1.32
*Actinomyces*	*A. odontolyticus*	5.14 × 10^−5^	42.00	1.23
*T. denticola* and rel.	*T. denticola*	2.31 × 10^−8^	34.89	1.50
*Eikenella*	*E. corrodens*	2.17 × 10^−6^	14.85	1.36
*H. parainfluenzae* and rel.	*H. parainfluenzae*	2.87 × 10^−4^	44.89	1.17
*A. actinomycetemcomitans*	*A. actinomycetemcomitans*	4.77 × 10^−5^	14.02	1.34
*Campylobacter*	*C. sputorum subsp. sputorum*	3.43 × 10^−5^	40.55	1.32
*Capnocytophaga*	*C. sputigena*	3.60 × 10^−4^	22.72	1.09
*P. gingivalis*	*P. gingivalis*	1.36 × 10^−6^	30.22	1.15
*P. denticola* and rel.	*P. denticola*	3.86 × 10^−5^	25.14	1.34
*P. intermedia* and rel.	*P. intermedia*	2.37 × 10^−7^	26.10	1.33
*P. melaninogenica* and rel.	*P. melaninogenica*	1.09 × 10^−6^	17.57	1.22

With the purpose to evaluate the reliability of the OralArray, each 16 S rRNA amplicon was subjected to at least four independent LDR-UA experiments, and all replicates gave the correct detection (data not shown). Hence, the OralArray showed a complete reproducibility (100%) on 16 S amplicons prepared from oral target bacteria.

**Figure 2:**
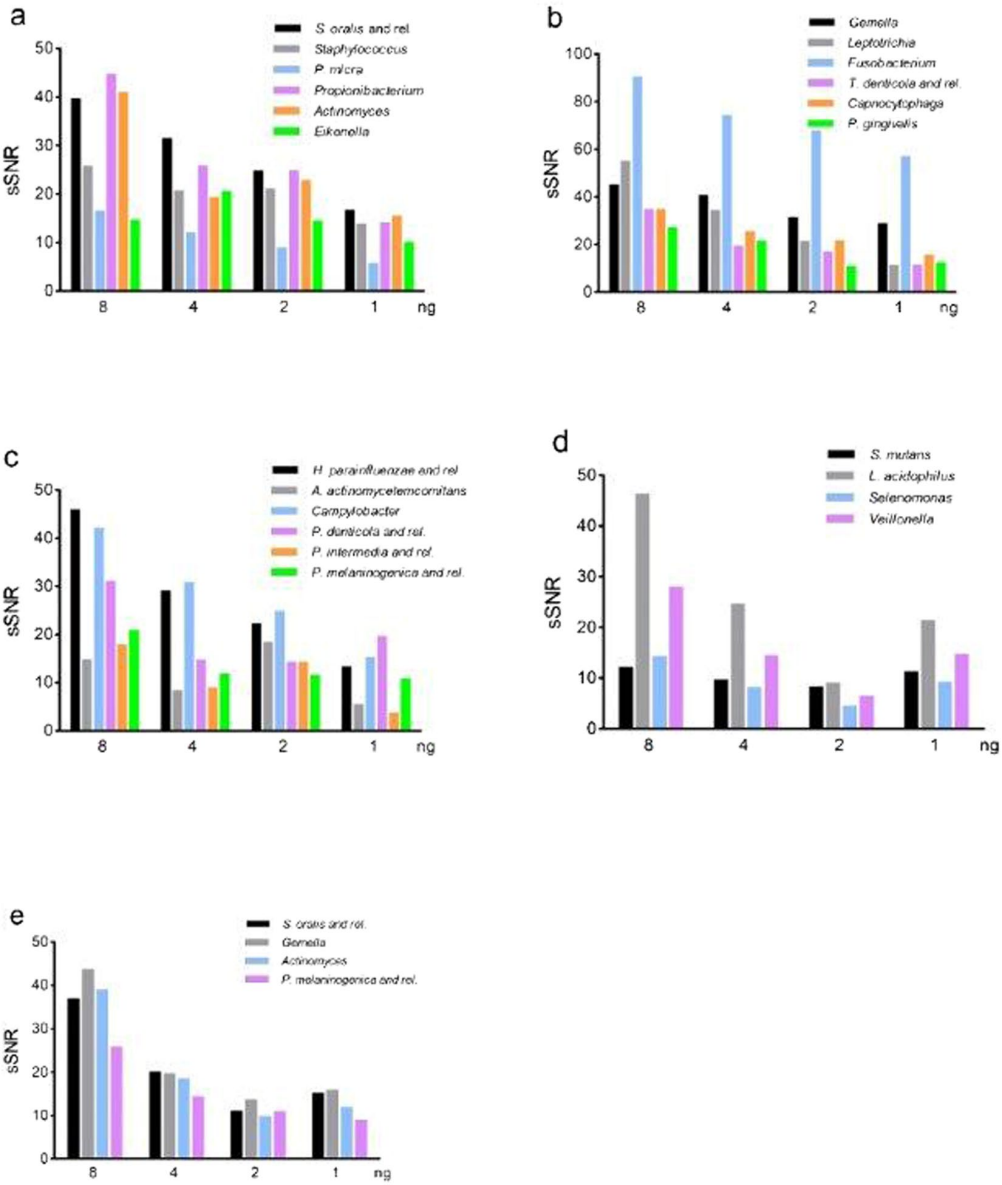
Sensitivity of the OralArray on DNA mixes. Artificial mixes of 16 S rRNA amplicons were subjected to LDR-UA analysis, sSNR values were plotted vs ng of each DNA amplicon. sSNR values were obtained with decreasing amounts of DNA mixes containing: (**a**) *S. oralis, S. aureus, P. micra, P. acidifaciens, A. odontolyticus*, and *E. corrodens* 16 S rRNA amplicons (8–1 ng each); (**b**) *G. morbillorum, L. buccalis, F. nucleatum, T. denticola, C. sputigena*, and *P. gingivalis* 16 S rRNA amplicons (8–1 ng each); (**c**) *H. parainfluenzae, A. actinomycetemcomitans, C. sputorum, P. denticola, P. intermedia*, and *P. melaninogenica* 16 S rRNA amplicons (8–1 ng each); (**d**) *S. mutans, L. acidophilus, S. noxia*, and *V. parvula* 16 S rRNA amplicons (8–1 ng each); (**e**) *S. oralis, G. morbillorum, A. odontolyticus*, and *P. melaninogenica* 16 S rRNA amplicons (8–1 ng each) added with human genomic DNA up to 100 ng of total DNA (68–96 ng).

In order to define the detection limit of the OralArray, LDR-UA experiments were carried out with decreasing amounts of 16 S rRNA amplicons, in the range from 8 to 1 ng (corresponding to 5.6–0.7 fmol, respectively). All amplicons were correctly detected showing P-values below 0.005, even at the lowest amount, thus defining the sensitivity limit of the tool to 1 ng, which corresponds to 0.7 fmol of PCR product. Notably, sSNR values decreased along with gradually reducing template concentration, showing an average Pearson correlation coefficient of 0.84 ± 0.20. In addition, sensitivity experiments were carried out on artificial mixes of 16 S rRNA amplicons, containing equal amounts of PCR amplicons obtained from six/four members of the human oral microbiota. All targets were specifically recognized even at the lowest amount, confirming the low sensitivity limit of the OralArray. Even in these DNA mixes, a linear correlation between sSNR values and 16 S rRNA amplicon amounts was observed (Fig. 2a–d). In order to evaluate the potential interference of human DNA with the OralArray analysis, a set of LDR-UA experiments was performed in the presence of human genomic DNA. Also in these experimental conditions, the targets were correctly detected and sSNR values correlated with DNA amounts (average Pearson correlation coefficient 0.97 ± 0.02) (Fig. 2e).

Given the good linear correlation between sSNR values and 16 S rRNA amplicon amounts, the OralArray could be potentially employed for semi-quantitative analysis or relative quantification. The average ratio between sSNR and DNA amount was used to estimate probe efficiency. Efficiency coefficients were, then, employed to scale IF data.

### Validation of the OralArray on clinical oral samples

**Figure 3:**
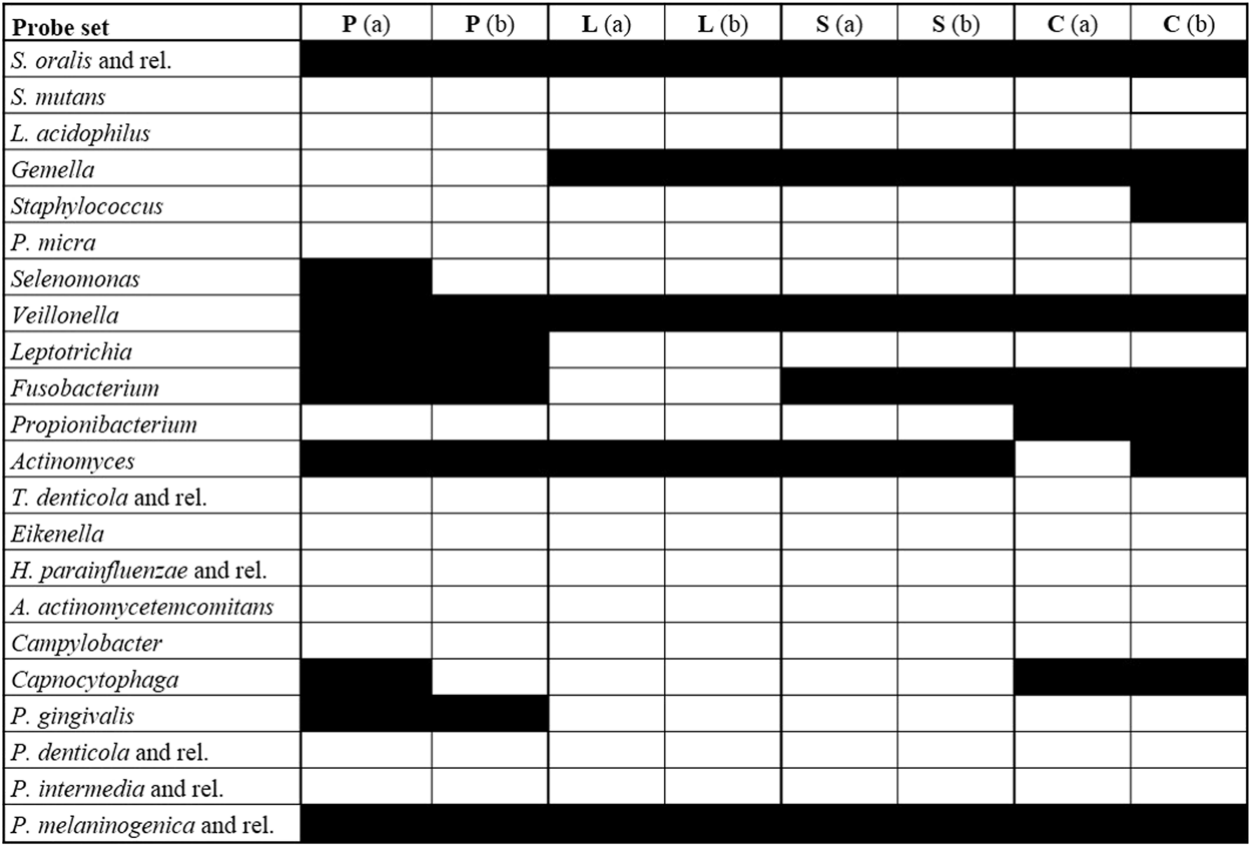
Reproducibility of the OralArray analysis on oral samples. Four samples, representative of four different oral sites (S: saliva, L: lingual plaque, P: supragingival plaque, C: healing cap), were subjected to two separate LDR-UA experiments (**a,b**). Response of each probe set for the presence/absence of the bacterial target is displayed: black: positive response (P < 0.01); white: negative response (P > 0.01).

The new tool OralArray was applied to investigate the bacterial signature of thirty-six oral specimens collected from ten patients at four different oral sites, i. e. saliva, lingual plaque, supragingival plaque, and healing cap. For five patients (namely P1 to P5), samples of saliva, lingual plaque, supragingival plaque were collected; for the other five patients (P6 to P10), samples corresponding to healing caps were also available. For all samples, total DNA was extracted, the bacterial 16 S rRNA gene was amplified and analysed by LDR-UA. Firstly, four samples, representative of the four different oral sites, were subjected to two separate LDR-UA experiments, in order to establish the reproducibility of the analysis on clinical samples. For each experiment, a profile of presence/absence of probe response was obtained, considering a threshold value P = 0.01 for significance. The OralArray has proved to be applicable to oral samples of different origin, and showed a reproducibility of 97.7% (calculated as the percentage of the probes giving the same response in the technical replicates). Indeed, the cluster analysis of the microbial profile obtained with OralArray showed that replicates clustered together, showing very high cluster stability values ranging from 85 to 100 (Fig. 3, Supplementary Fig. S1).

**Figure 4:**
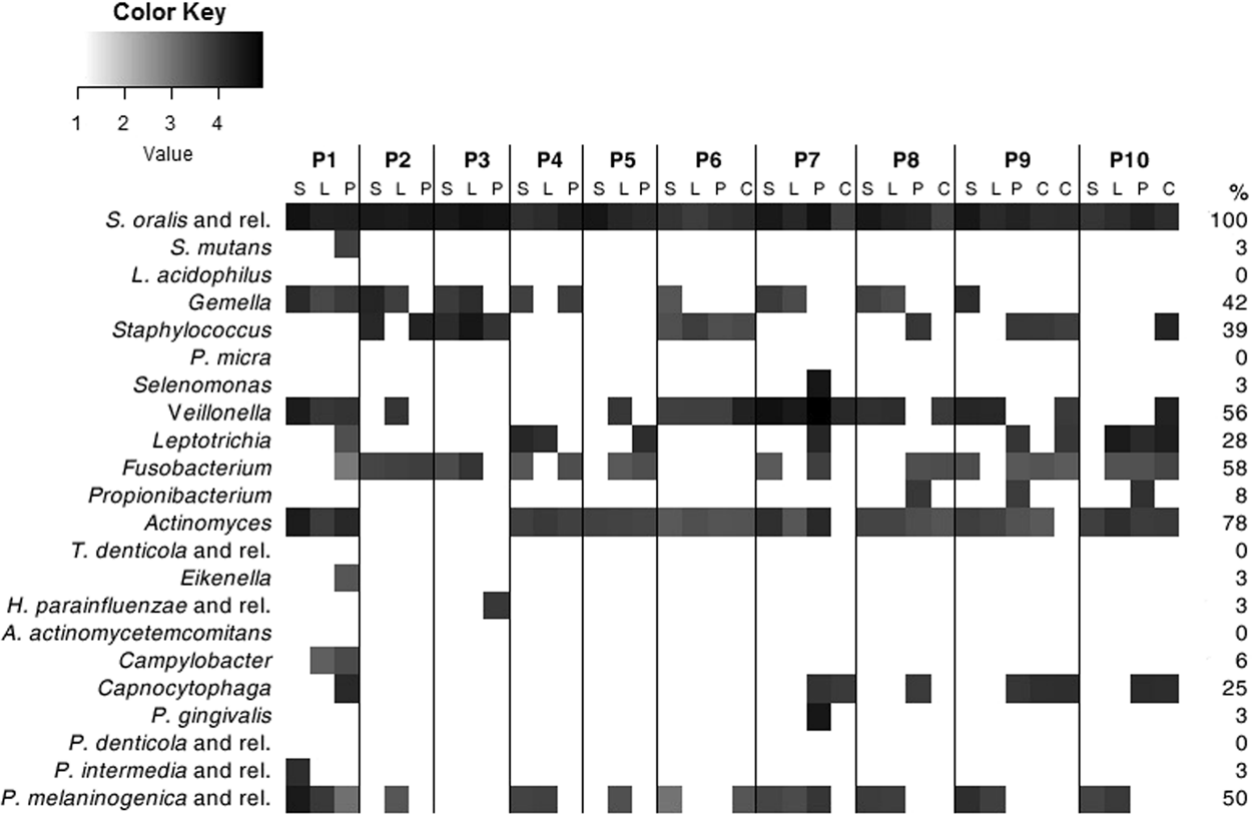
Heatmap of the OralArray data. Samples were collected from ten subjects (P1-P10) at four oral sites (S: saliva, L: lingual plaque, P: supragingival plaque, C: healing cap) and analyzed by OralArray. Fluorescence intensities (IFs) were normalized over the IFs of the Zip Code 63, as described in the Methods section. The mean values of the logarithm of IFs (n = 4) for each bacterial target are plotted in a scale of grey, by using R “made4” package. Prevalence of bacterial targets in the oral samples are also reported, expressed in percentage (%).

Subsequently, the analysis was conducted on the entire set of samples and results are visualized in Fig. 4. *Streptococcus oralis* (or related species) was ubiquitously detected in all specimens, the genus *Actinomyces* was the second most common bacterial target, detected by the OralArray in 78% of the samples. The genera *Fusobacterium* and *Veillonella* were, also, very frequently identified (58% and 56% of the samples, respectively), as well as *Prevotella melaninogenica* (or related species), present in half of the samples. *Gemella* and *Staphylococcus* genera were, also, quite common in the oral samples, being positive in 42% and 39% of the specimens, respectively; on the other hand, *Leptotrichia* and *Capnocytophaga* genera were found in 28% and 25% of the samples analysed. The bacterial targets *S. mutans, Selenomonas, Propionibacterium, Eikenella, H. parainfluenzae* (or related species), *Campylobacter, P. gingivalis*, and *P. intermedia* were only seldom detected (3–8%). Finally, *L. acidophilus, P. micra, T. denticola* (and related species), *A. actinomycetemcomitans*, and *P. denticola* were never found in the analysed samples.

**Figure 5:**
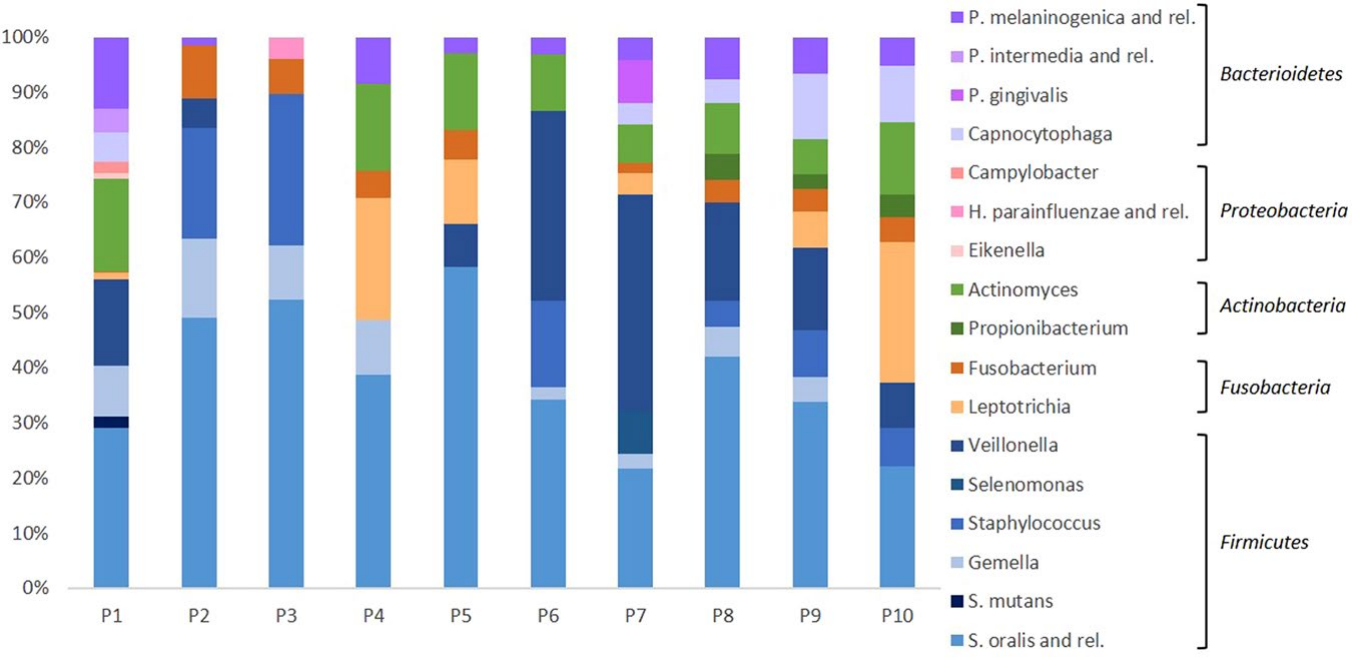
Relative abundance of bacterial targets detected by the OralArray, grouped for individual. For each individual (P1-P10), fluorescence intensities of saliva, lingual plaque, supragingival plaque, and healing cap were grouped and average relative contributions were calculated as percentage of the total fluorescence intensity.

In order to search for possible correlations between oral microbiota composition and individual or sample type (oral site of collection), the entire set of quantitative IF data collected from OralArray were subjected to analysis of variance (ANOVA). We, firstly, sorted samples on the basis of the subject, grouping together saliva, lingual plaque, supragingival plaque, and healing cap data of each patients, and we found that the microbial signature was significantly different in relation to the subject considered (P = 0.0273). When the statistic test was applied only to saliva, lingual plaque, and supragingival plaque data, the differences in the microbiota between individuals was highly significant (repeated measures ANOVA, P < 0.0001). On the contrary, the oral site of collection did not significantly affect the bacterial community composition. Indeed, when we grouped data on the basis of the sample type (saliva, lingual plaque, supragingival plaque, and healing cap) we found a high variability (P = 0.0582). Thus, samples collected from the same individual were sorted together and average relative abundance was calculated (Fig. 5). As expected, all subjects were quantitatively dominated by *Firmicutes*, with an average relative abundance of 67.7% ± 17.5%. Notably, *Firmicutes* exceeded 80% of abundance in P2, P3, and P6 subjects. In particular, *S. oralis* (and related species) was the highest signal in eight out of ten individuals, whereas *Veillonella* was predominant in P6 and P7 samples. Bacterial targets belonging to the phylum *Fusobacteria* were detected in the oral microbiota of nine individuals, with variable relative abundance, ranging from 30% to 1.5%; *Leptotrichia* signal was predominant in six individuals, whereas, in the other three, only *Fusobacterium* was detected (P2, P3, and P8). Members of the genus *Actinomyces* (phyum *Actinobacteria*) were retrieved in eight individuals, three of whom were, also, characterised by the presence of *Propionibacterium* (P8, P9, and P10). Representatives of the phylum *Bacterioidetes* were found in the oral microbiota of nine subjects (all subjects except P3), all being positive for *P. melaninogenica* (or related species) and five to *Capnocytophaga*; the bacterial targets *P. intermedia* (or related species) and *P. gingivalis*, on the other hand, were detected only in one individual (P1 and P7, respectively). Finally, evidence of a presence of microorganisms belonging to the *Proteobacteria* phylum was found only in the oral samples of two individuals (P1 and P3): P1 was colonised by *Eikenella* and *Campylobacter* genera, P3 by *H. parainfluenzae* (or related species).

Concerning the oral site of sample collection, although it did not significantly affect the bacterial community composition, it is worth to notice that some bacterial targets were preferentially or exclusively detected in a particular oral sample type. *Gemella* and *P. melaninogenica* (and related species) were prevalently found in saliva and lingual specimens rather than in plaque and healing caps. Indeed, *Gemella* was detected in eight saliva samples out of ten, and in half of lingual samples, but only in two plaque specimens and never in healing caps. Analogously, *P. melaninogenica* was found positive in seven saliva and eight lingual samples out of ten, while only in two plaques out of ten, and one healing cap out of six. Bacteria of the genus *Capnocytophaga* were detected only in samples collected from supragingival plaque (five out of ten) and healing caps (four out of six). In general, supragingival plaque was characterised by the largest variety of bacterial targets, indeed *S. mutans, Selenomonas, Propionibacterium, Eikenella, H. parainfluenzae* (or related species), and *P. gingivalis* were detected only in plaque samples.

### Application of the OralArray to evaluate the effects of chlorhexidine treatment to healing caps before the usage

**Figure 6:**
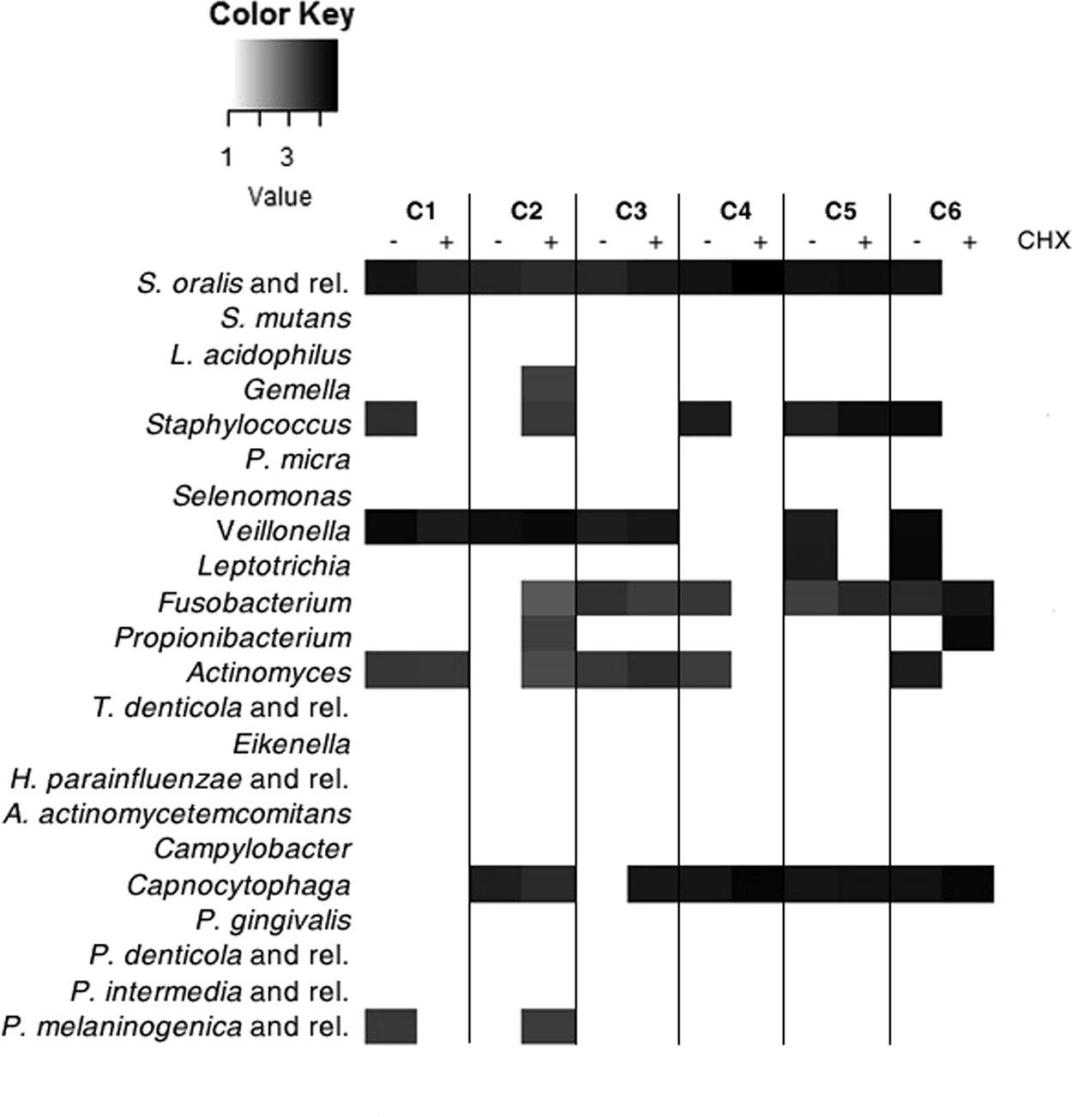
Heatmap of the OralArray data of control and chlorhexidine treated healing caps. Control and chlorhexidine-treated healing caps were put on the implants for two weeks, then collected, and bacterial signature depicted by OralArray. Fluorescence intensities (IFs) were normalized over the IFs of the Zip Code 63, as described in the Methods section. The mean values of the logarithm of IFs (n = 4) for each bacterial target are plotted in a scale of grey, by using R “made4” package. C1-C6 referred to healing cap pairs. CHX: chlorhexidine.

Given the good performance of the OralArray on oral clinical samples, the tool was employed to evaluate the effects of a disinfectant treatment on healing caps before their usage. For this purpose, patients under treatment for a prosthetic rehabilitation on at least two implants were enrolled. One implant was sealed with a sterile healing cap, the other one with a sterile healing cap previously immersed in chlorhexidine 0.2% solution. Healing caps were collected after two weeks, total bacterial DNA was isolated and amplified, and successively analysed by OralArray. Six pairs of healing caps were considered (C1-C6), and the bacterial community composition of paired control and chlorhexidine-treated healing caps was compared (Fig. 6). For C1, C4, C5, C6, the treatment with the disinfectant resulted in a reduced microbial complexity, in terms of number of bacterial targets detected. Indeed, in C1 chlorhexidine treatment caused a reduction of complexity from five to three bacterial targets, in C4 from five to two, in C5 from six to four, and in C6 from seven to three. On the other hand, chlorhexidine treatment had opposite effect on the microbial complexity of C2 and C3 healing caps pairs, which shifted from three to nine, and four to five, respectively. In addition, no correlation was found between the IF intensity registered by the OralArray and the chlorhexidine treatment, indicating no quantitative changes in bacterial populations induced by chlorhexidine.

## Discussion

The quali-quantitative characterization of the oral microbiota is crucial for an exhaustive knowledge of the oral ecology and the modifications of the microbial composition that occur during the most common periodontal pathologies. Among these, perimplant mucositis and perimplantitis are emerging problems related to the increasing use of dental implants, negatively affecting the success rate of the prosthetic rehabilitation^39^.

In the present study, we described the development and validation of a phylogenetic DNA-microarray specifically designed for the oral microbiota (OralArray), capable to detect the most representative bacterial groups that colonize the oral cavity in healthy and pathological conditions. Our array is based on the ligation detection reaction (LDR), a molecular assay which possesses inherent advantages over microarrays whose discriminative power is based on hybridization. Ligation-based assays are based on a Pfu thermostable DNA ligase that seals the nick between the “discriminating” and the “common” probes only when there is a perfect match between the two and the template. Therefore a single mismatch in 3′ terminal position of the discriminating probe is enough to prevent ligation^40^. Other advantages of the LDR-UA format are less problems during hybridization due to the secondary structures of the target DNA and steric hindrances and the fact that all Zip Codes have been designed in order to have the same melting temperature and do not need further optimization in the hybridization step^25^. LDR-UA, thus, allows high performance in terms of sensitivity and discriminatory power and combines the high throughput of microarrays with the versatility of the PCR.

Indeed, the OralArray includes probe pairs targeted to microbial species or genera that characterize the healthy mouth, as well as bacteria involved in dental caries, periodontal disease, and perimplantitis^3, 5, 32, 33^.

However, emerging data sustain the “ecological plaque hypothesis” to explain the etiology of the most prevalent oral diseases: such hypothesis ascribes the onset of an oral disease to a dysbiosis of the resident oral microflora, driven by an ecological perturbation, rather than the colonization of specific pathogen microbes responsible for particular infections^6, 11, 12, 41^. In this perspective, the detection/quantitation of a pool of putative ‘pathogenic’ microbial species should be addressed by molecular tools, in order to monitor microbiota compositional fluctuations for the correct diagnosis or susceptibility assessment of oral polymicrobial diseases.

The OralArray analysis has been proved highly specific, reproducible and sensitive. The tool was able to detect all tested oral-related 16 S rRNA amplicons without ambiguity, with a reproducibility of 100%. The detection limit, corresponding to 1 ng (0.7 fmol) of PCR product for all probe sets, was equal or lower than that calculated for other LDR-UA based DNA microarrays (HTF-MicroBi.Array: 0.7–75 fmol^25^; VaginArray: 6–12 ng^26^). Moreover, a major feature of the OralArray is the good linear correlation between IF signals (expressed as sSNR) and DNA template concentration, which enables the potential employment of the tool for semi-quantitative analysis and relative quantification. However, it must be stressed that the main goal of our phylogenetic array is the representation of the relative abundance of selected dominant bacteria within a complex ecosystem, such as the oral cavity.

Although the advent of next-generation sequencing techniques in microbial ecology is allowing the detection of the full bacterial community of human microflora (including oral) at reasonable costs, the OralAray permits a faster and cheaper detection of the most important bacterial groups in samples from oral sites. Each LDR-UA experiment costs down to about 6 euros per sample, including array production, probe synthesis and ligation, and takes about 7 hours to be performed, including ligation, hybridization on UA and scanner detection. NGS techniques, on the other hand, can be useful to discover new potentially interesting targets to be addressed; thanks to the flexibility of the LDR-UA, the platform can be easily implemented with up to a total of forty-seven probeset per array in the current configuration.

The OralArray has been applied to oral samples collected from different oral sites, as well as healing caps, and microbial signatures were successfully obtained from all different oral samples.

It has been reported that healthy oral microbiota is characterized by a conserved microbial fingerprint at the genus level, but large inter-individual differences were also evidenced, especially at species and strain level^9, 42^. In addition, similarities between microbial communities detected on teeth, tongue samples, and implants collected from the same individual were found^43, 44^. Our data strengthened the presence of an individual oral microbial profile, given that the detected microbial panel is significantly different in relation to the subject analysed.

We failed to demonstrate a statistic correlation between the bacterial communities composition and the oral site of sample collection. Nevertheless, some bacterial targets were prevalent in particular oral sample types, and our findings are in agreement with data previously reported in literature obtained by NGS^45^. These authors delineated the core bacterial taxa in the oral cavity from over two-hundred individuals participating in the Human Microbiome Project, reporting that saliva and lingual plaque core microbiomes include *Prevotella* species, and supragingival plaques are highly colonised by *Capnocytophaga*. The concordance of our findings with the NGS data already published represents a further element that supports the validity of our array as a phylogenetic tool.

The OralArray was, also, applied to evaluate the efficacy of a disinfectant treatment on the healing caps before their usage: no significant differences in the microbial community composition were noticed between control and disinfectant-treated healing caps.

In conclusion, in the present paper, we reported the development and validation of a new phylogenetic tool specifically designed for the oral microbiota. We demonstrated that the OralArray is able to efficiently and quickly delineate the core bacterial signature of various oral sample types, including healing caps, and can be easily applied in clinical trials.

## Methods

### Selection of bacterial targets and consensus extraction

Bacterial genera, cluster of phylogenetically related species or single species representative of the human oral ecosystem were rationally selected on the basis of the available literature^3, 5, 30–34^. For such bacteria, 16 S rRNA gene sequences were retrieved from Ribosomal Database Project^46^ (RDP, http://rdp.cme.msu.edu/) (named ‘positive set’) and group-specific consensus sequences were extracted with a cut-off of 75% for base calling (i.e.: for each position, the consensus reports the nucleotide present in at least ¾ of the positive set sequences). Nucleotides which occurred at lower frequencies were replaced by the appropriate IUPAC ambiguity code. Analogously, for each target of the array, a “negative set” of 16 S rRNA sequences was built, which includes sequences each probe set should not target.

### Design of common and discriminant probes

Selected sequences were subjected to multiple alignments by ClustalW^47^. All the LDR probe pairs were designed by using ORMA^35^ following the procedure used for the HTF-MicroBi.Array^25^. Briefly, for each target, a DP and a CP were designed on the appropriate consensus sequence, identifying a region capable to discriminate sequences belonging to positive and negative sets. Both DP and CP were required to be between 25 and 60 bases pair, with a melting temperature (Tm) of 68 ± 1 °C, and with maximum 4 degenerated bases. A cZip Code was added at the 3′ end of each CP, a Cy3 label was attached to the 5′ end of each DP. Specificity and coverage of each candidate probe was assessed *in silico* using the tool Probe Match of the RDP database. The probe pairs were required to perfectly match the sequences of the positive set and to possess at least one mismatch at the 3′ end of the discriminating probe respect to the entire negative set.

### Bacterial genomic DNA

Genomic DNA from *Streptococcus agalactiae* DSM2134, *Streptococcus mutans* DSM20523, *Streptococcus oralis* DSM20627, *Gemella morbillorum* DSM20572, *Selenomonas noxia* DSM19578, *Veillonella parvula* DSM2008, *Parvimonas micra* DSM20468, *Actinomyces odontolyticus* DSM19120, *Propionibacterium acidifaciens* DSM21887, *Eikenella corrodens* DSM8340, *Campylobacter sputorum* subsp. *sputorum* DSM10535, *Haemophilus parainfluenzae* DSM8978, *Aggregatibacter actinomycetemcomitans* DSM8324, *Prevotella denticola* DSM20614, *Prevotella intermedia* DSM20706, *Prevotella melaninogenica* DSM7089, *Porphyromonas gingivalis* DSM20709, *Capnocytophaga sputigena* DSM7273, *Fusobacterium nucleatum* subsp. *polymorphum* DSM20482, *Leptotrichia buccalis* DSM1135, and *Treponema denticola* DSM14222 was directly obtained from the DSMZ (Braunschweig, Germany).

Bacterial DNA from *Lactobacillus acidophilus* DSM20079 and *Staphylococcus aureus* ATCC12600 was extracted from 10^9^ bacterial cells by using the DNeasy Blood and Tissue Kit (Qiagen, Düsseldorf, Germany) following the manufacturer instructions. *L. acidophilus* was grown on De Man-Rogosa-Sharpe (MRS) broth with cysteine (0.5 g/L) at 37 °C, under an anaerobic atmosphere (Anaerocult, Merck, Darmstadt, Germany). *S. aureus* ATCC12600 was grown at 37 °C aerobically on Luria-Bertani (LB) broth.

### Collection of oral samples

Oral samples were collected in the field of routine clinical practice in the Dental Clinic ASST Santi Paolo e Carlo, University of Milan (Milan, Italy). The ethical board of the ASST Santi Paolo e Carlo gave the approval for this study (2016/ST/085). The patients enrolled had to understand and sign a voluntary informed consent. The methods were performed in accordance with the approved guidelines. Systemic exclusion criteria were: medical conditions requiring prolonged use of steroids, severe hemophilia, bisphosphonate medication, history of leukocyte dysfunction and deficiencies, history of head and neck radiation or chemotherapy, history of renal failure, history of uncontrolled endocrine disorders, physical handicaps interfering with ability to perform adequate oral hygiene, alcoholism or drug abuse, HIV infection, smoking >10 cigarettes or cigar equivalents per day. Local exclusion criteria were: local inflammation, including untreated periodontitis, mucosal diseases such as erosive lichen planus, history of local irradiation therapy, persistent intraoral infection, patients with inadequate oral hygiene or unmotivated for adequate home care. Patients had not received any antimicrobial treatment in the three months before the enrollment. A total of ten patients were included (six females, four males, mean age 46.7 years). Among these, five patients were under treatment for a prosthetic rehabilitation on at least 2 Straumann RN implants (Insitute Straumann AG, Basel, Switzerland). The oral samples were collected as follows: 1) non-stimulated saliva: the patient was asked to collect his non-stimulated saliva over a period of 5 min in a sterile tube; 2) lingual plaque: a gentle air flow was used to remove superficial saliva from the tongue, then a sterile cotton pellet was brushed on the tongue for 30 s and, then, released in a sterile tube filled with 1 mL of saline solution; 3) supragingival plaque: a gentle air flow was used to remove superficial saliva from the teeth, then, a sterile cotton pellet was brushed on dental surfaces of posterior elements for 30 s and, subsequently, released in a sterile tube filled with 1 mL of saline solution; 4) implant healing caps: one implant was washed with saline solution internal irrigation for 30 s and, then, dried off with a gentle blow of air; the corresponding healing cap was immersed in saline solution for one minute just before tightening on the implant. The other implant was washed with 0.2% chlorhexidine solution internal irrigation, then dried off, and the healing cap was immersed in 0.2% chlorhexidine solution before tightening on the implant. After two weeks, healing caps were collected in sterile tubes filled with 1 mL of saline solution. All tubes were sealed, and stored at −80 °C immediately after the sample collection.

### Extraction of genomic DNA from oral samples

Total bacterial DNA was extracted from oral samples as described by Cruciani *et al*.^48^ with slight modifications: 0.5–1 mL of each specimen was centrifuged at 10,000 g for 15 min and the pellet was directly resuspended in 180 μL of enzymatic lysis buffer (20 mM Tris-HCl pH 8, 2 mM EDTA, 1.2% Triton X-100, 20 mg/mL lysozyme) and incubated at 37 °C for 30 min. 200 mg of glass beads were added and the sample was mixed by vortexing for 1 min. Total DNA was extracted by using the DNeasy Blood and Tissue kit following the protocol “Pretreatment for Gram-positive bacteria”. DNA was quantified by using NanoDrop ND-1000 (Thermo Scientific, Wilmington, DE).

### PCR conditions

All the oligonucleotide probes were synthesized by Thermo Fisher Scientific (Ulm, Germany). 16 S rRNA gene was amplified by using a Biometra Thermal Cycler II (Biometra, Gottingen, Germany) and 2.5 U of GoTaq Flexi polymerase (Promega, Madison, WI) in a final volume of 50 μL following the protocol described in Cruciani *et al*.^26^. PCR products were purified by using a High Pure PCR Clean up Micro kit (Roche, Mannheim, Germany), following the manufacturer instructions, and quantified by NanoDrop ND-1000.

### LDR-UA conditions

The experimental procedure for both the chemical treatment and the spotting of UA is described in detail in Consolandi *et al*.^49^. Briefly, each UA consists of synthetic oligonucleotides, called Zip Codes, each printed in quadruplicate within the printing area; two Zip Codes, namely 63 and 66, act as ligation and hybridization controls, respectively; pure printing buffer was used as a negative control (blank). Each printed slide contains 8 UAs, each being a 13 × 16 matrix of a total of 208 spots of nominal diameter size of about 120–150 μm (Supplementary Fig. S2). Ligase Detection Reactions (LDRs) were carried out in a final volume of 20 μL containing 250 fmol of each specific probe (CP and DP), 25 fmol of a synthetic template (5′-AGCCGCGAACACCACGATCGACCGGCGCGCGCAGCTGCAGCTTGCTCATG-3′, recognized by a specific set of probes bearing cZip Code 63), 4 U of *Pfu* DNA ligase (Agilent Technologies, Palo Alto, CA) and appropriate amount of PCR product. In specificity tests 10 ng of PCR product were used. Three different sensitivity tests were performed as follows: a) decreasing amounts from 8 ng to 1 ng of single PCR product, b) decreasing amounts from 48 ng to 6 ng of PCR product mixes composed by six different 16 S RNA amplicons or decreasing amounts from 24 ng to 4 ng of PCR product mixes composed by four different 16 S RNA amplicons, c) decreasing amounts from 24 ng to 4 ng of PCR product mixes composed by four different 16 S RNA amplicons added with increasing amounts of human genomic DNA up to 100 ng of total DNA content. In human oral samples analyses, 48 ng of PCR product were used. Prior to LDR, the DNA was denatured at 95 °C for 5 min; then, the reaction was cycled for 30 rounds at 94 °C for 30 s and at 60 °C for 4 min, in a Biometra Thermal Cycler II (Biometra). Before hybridization, LDR mixtures were diluted as described in Castiglioni *et al*.^28^ and 100 fmol of cZip 66 oligonucleotide (complementary to Zip Code 66, 5′-Cy3-GTTACCGCTGGTGCTGCCGCCGGTA-3′) plus 6 ng of salmon testes DNA were added. Zip Code 66 (hybridization control) and Zip Code 63 (ligation control) were used for locating the sub-matrixes during the scanning and for normalization purposes, respectively.

### UA scanning and data analysis

Arrays were scanned by using a ScanArray 5000 scanner (Perkin Elmer Life Sciences, Boston, MA) at 10 µm resolution and the fluorescence intensity (IF) was quantitated by ScanArray Express 3.0 software, as previously described^26^. IFs were subjected to a normalization procedure based on the IFs of the Zip Code 63, which is added to LDR mixes in fixed amounts, applied as follows: (a) outlier values (2.5-fold above or below the average) were discarded; (b) a correction factor was calculated in order to set the average IF of Zip Code 63 to 50000 (n = 6); (c) the correction factor was applied to both the probe spots and background IF values. Significantly present spots were determined using a one-tailed *t*-test comparing, for each Zip Code, the distribution of IFs along all replicates (n = 4) with the distribution of IFs of negative controls (i.e.: “Blanks”, where only printing buffer has been spotted) (n = 6). Eventual outliers (those spots showing an IF 2.5-fold above or below the average) were discarded before performing the test. A P value = 0.01 was considered as a threshold for significance. The signal-to-noise ratio (SNR) was calculated as the ratio between the average IF of each probe and the average IF of Blank spots.

### Statistical analysis

Statistical analysis was performed by using GraphPad software (GraphPad software Inc., San Diego, CA). Hierarchical clustering of LDR-UA detection signals (a 0/1 matrix where “1” means a positive signal for the corresponding probe, a “0” otherwise) was performed using Pearson correlation as metric and average linkage. Cluster stability was determined in R (v 2.13.2) by pvclust package (v 2.0.0)^50^.

## Electronic supplementary material

Supplementary information
