# Supplementary material for: Design and validation of a DNA-microarray for phylogenetic analysis of bacterial communities in different oral samples and dental implants
Source: Sci Rep. 2017 Jul 24;7:6280. doi: 10.1038/s41598-017-06743-6 (PMC5524749; doi:10.1038/s41598-017-06743-6)
Supplement: Supplementary file 1 — Supplementary information [file 41598_2017_6743_MOESM1_ESM.pdf]

**Design and validation of a DNA-microarray for phylogenetic analysis of bacterial communities in different oral samples and dental implants**

Carola Parolin, Barbara Giordani, Rogers Alberto Ñahui Palomino, Elena Biagi, Marco Severgnini, Clarissa Consolandi, Giada Caredda, Stefano Storelli, Laura Strohmenger, and Beatrice Vitali

**Supplementary Table S1: Probe sets included in the OralArray.** Nucleotide sequences, lengths (Len), melting temperature (Tm) and number of degenerated bases (#Deg bases) of discriminating (DP) and common probes (CP).

| Probe set | Discriminating probe (DP) | Common probe (CP) | Len DP | Len CP | Tm DP | Tm CP | #Deg bases DP | #Deg bases CP |
|---|---|---|---|---|---|---|---|---|
| ***Streptococcus oralis* and rel** | GTGGCTTAACCATAGTACGCTTTGGAAACTGTTTAACTTGAGTGCAA | GAGGGGAGAGTGGAATTCCATGTGTAGCGGTGAAATG | 47 | 37 | 67.2 | 67.8 | 0 | 0 |
| ***Streptococcus mutans*** | CTAGTAGATGGACCTGCGTTGTATTAGCTAGTTGGTAAGGTAAGA | GCTTACCAAGGCGACGATACATAGCCGACCTGAG | 45 | 34 | 67.3 | 68.0 | 0 | 0 |
| ***Gemella*** | CAAGTCTTGACATACTGTGAGGACACAAGAGATTGTGTTGTTCTG | ACCTTTGGTTAGACACAGATACAGGTGGTGCATGGTTGTC | 45 | 40 | 67.3 | 67.6 | 0 | 0 |

| | | | | | | | | |
|---|---|---|---|---|---|---|---|---|
| ***Parvimonas micra*** | GCGAAGAAGGTTTTCGAATCG TAAAGCTCTGTCCTATGAGAA GAT | AATGACGGTATCATAGGAGGA AGCCCCGGCTAAATACG | 45 | 38 | 67.3 | 67.7 | 0 | 0 |
| ***Selenomonas*** | GGSCTTGACATTGASTGAAAGA GCTAGAGATAGCTCCC | TCTCTTCGGAGACASGAAAAC AGGTGGTGCATGGC | 38 | 35 | 67.7 | 67.9 | 2 | 1 |
| ***Veillonella*** | GACCTGGGCTACACACGTACT ACAATGGGAGTTAATAGAC | GGAAGCGARAYCGCGAGRTGG AGCAAACCC | 40 | 30 | 67.6 | 67.8 | 0 | 3 |
| ***Propionibacterium*** | CTTGAGGAAGGTAGGGGAGAA TGGAATTCCTGGTGG | AGCGGTGGAATGCGCAGATAT CAGGAGGAACACC | 36 | 34 | 67.9 | 68.0 | 0 | 0 |
| ***Actynomices*** | CTGGCTTAACTGGGGGCKTGC GGTGGGT | ACGGGCNGGCTTGAGTGCGGT AGGGG | 28 | 26 | 68.0 | 68.2 | 1 | 1 |
| ***Treponema denticola* and rel** | GTCCAGGGCTACACACGTGCT ACAATGGTTGCTACAAAT | CGAAGCGACACCGCGAGGTCA AGCAAAACG | 39 | 30 | 67.6 | 67.1 | 0 | 0 |
| ***Eikenella*** | CGCAAGACCTCGCGTTATTCGA GCGGCCGATAA | CTGATTAGCTAGTTGGTGGGGT AAAGGCCTACCAAGG | 33 | 37 | 68.1 | 67.8 | 0 | 0 |
| ***Haemophilus parainfluenzae* and rel** | GTTGGTGAGGTAAAGGCTCAC CAAGCCGACGATCT | CTAGCTGGTCTGAGAGGATGA CCAGCCACACTG | 35 | 33 | 67.9 | 68.1 | 0 | 0 |

| | | | | | | | | | |
|---|---|---|---|---|---|---|---|---|---|
| ***Aggregatibacter actinomycetemcomitans*** | GGCTTAACCTGGGAATTGCATTTCATACTGGGGGTCTG | GAGTACTTTAGGGAGGGGTAGAATTCCACGTGTAGCG | 38 | 37 | 67.7 | 67.8 | 0 | 0 |
| ***Capnocytophaga*** | GAGGCAGCAGTGAGGAATATTGGACAATGGTCGGAAGA | CTGATCCAGCCATGCCGCGTGCAGGATG | 38 | 28 | 67.7 | 67.2 | 0 | 0 |
| ***Porphyromonas gingivalis*** | GGTGCGTAGGTTGTTCGGTAAGTCAGCGGTGAAAC | CTGAGCGCTCAACGTTCAGCCTGCCGTTGAAAC | 35 | 33 | 67.9 | 68.1 | 0 | 0 |
| ***Prevotella denticola* and rel** | CGTCGTGTAGCGGTGAAATGCTTAGATATGACGAAGAACC | CCGATTGCGAAGGCAGCCGGCGGGA | 40 | 25 | 67.6 | 67.5 | 0 | 0 |
| ***Prevotella intermedia* and rel** | CGCGTATCCAACCTTCCCTYYACTYGGGGATAC | CCCGTTGAAAGACGGCCTAATACCCGATGTTGTYC | 33 | 35 | 67.5 | 67.4 | 3 | 1 |
| ***Prevotella melaninogenica* and rel** | GCAGCGCATACTGGTTTCCTTGAGTACGCACAACGTT | GGCGGAATTCGTCGTGTAGCGGTGAAATGCTTAGATATG | 37 | 39 | 67.8 | 67.6 | 0 | 0 |

**Supplementary Table S2: List of 16S sequences used to build the phylogenetic tree reported in Figure 1.** Sequence name, Genbank ID and Length (bp) of 383 nucleotide sequences included in the phylogenetic analysis. Sequences have an average length of 1454.2±65.5 bp.

| **Sequence name** | **Genbank ID** | **Length (bp)** |
|---|---|---|
| *Actinomyces bovis* NCTC11535 | X81061 | 1434 |
| *Actinomyces bowdenii* M1956/95/1 | AJ234039 | 1513 |
| *Actinomyces canis* CCUG41706 | AJ243891 | 1428 |
| *Actinomyces cardiffensis* CCUG44997 | AJ421779 | 1522 |
| *Actinomyces catuli* CCUG41709 | AJ276805 | 1400 |
| *Actinomyces coleocanis* CCUG41708 | AJ249326 | 1437 |
| *Actinomyces dentalis* R18165 | AJ697609 | 1517 |
| *Actinomyces denticolens* NCTC11490 | X80412 | 1422 |
| *Actinomyces europaeus* CCUG32789A | Y08828 | 1410 |
| *Actinomyces funkei* CCUG42773 | AJ404889 | 1366 |
| *Actinomyces georgiae* DSM6843 | X80413 | 1422 |
| *Actinomyces gerencseriae* DSM6844 | X80414 | 1415 |
| *Actinomyces graevenitzii* CCUG27294 | AJ540309 | 1413 |
| *Actinomyces hominis* 7894GR | FJ617539 | 1502 |
| *Actinomyces hongkongensis* HKU8 | AF433168 | 1425 |
| *Actinomyces hordeovulneris* C.I.P.103149T | X82448 | 1426 |
| *Actinomyces howellii* NCTC11636 | X80411 | 1415 |
| *Actinomyces hyovaginalis* NCFB2983 | X69616 | 1496 |
| *Actinomyces israelii* C.I.P.103259T | X82450 | 1441 |
| *Actinomyces marimammalium* CCUG41710 | AJ276405 | 1416 |
| *Actinomyces massiliensis* 4401292 | EF558367 | 1481 |
| *Actinomyces meyeri* C.I.P.103148T | X82451 | 1441 |
| *Actinomyces naeslundii* NCTC10301 | X81062 | 1450 |

| | | |
|---|---|---|
| *Actinomyces nasicola* CCUG46092 | AJ508455 | 1475 |
| *Actinomyces neuii* DSM8576 | AM084228 | 1495 |
| *Actinomyces neuii* DSM8577T | AM084229 | 1497 |
| *Actinomyces odontolyticus* CCUG20536T | AJ234040 | 1412 |
| *Actinomyces oricola* CCUG46090 | AJ507295 | 1508 |
| *Actinomyces radicidentis* CCUG36733T | AJ251986 | 1401 |
| *Actinomyces radingae* APL1 | X78719 | 1429 |
| *Actinomyces ruminicola* B71 | DQ072005 | 1526 |
| *Actinomyces slackii* CCUG32792 | AJ234066 | 1435 |
| *Actinomyces suimastitidis* CCUG39276 | AJ277385 | 1416 |
| *Actinomyces timonensis* 7400942 | EU484334 | 1465 |
| *Actinomyces turicensis* APL10 | X78720 | 1453 |
| *Actinomyces urogenitalis* | AJ243791 | 1423 |
| *Actinomyces vaccimaxillae* R10176T | AJ427451 | 1493 |
| *Actinomyces viscosus* NCTC10951 | X82453 | 1417 |
| *Aggregatibacter actinomycetemcomitans* ATCC33384 | M75039 | 1484 |
| *Aggregatibacter actinomycetemcomitans* ANH9381 | CP003099 | 1541 |
| *Aggregatibacter actinomycetemcomitans* BM043 | GU413972 | 1513 |
| *Aggregatibacter actinomycetemcomitans* ChDCPV-A110 | KF933761 | 1504 |
| *Aggregatibacter actinomycetemcomitans* D11S-1 | CP001733 | 1485 |
| *Aggregatibacter actinomycetemcomitans* D7S-1 | CP003496 | 1540 |
| *Aggregatibacter actinomycetemcomitans* HG2864/104084 | FN547963 | 1201 |
| *Aggregatibacter actinomycetemcomitans* IDH392 | AB512011 | 1504 |
| *Aggregatibacter actinomycetemcomitans* JCM12985 | AB548673 | 1501 |
| *Aggregatibacter actinomycetemcomitans* JCM2434 | AB594758 | 1462 |
| *Aggregatibacter actinomycetemcomitans* JCM8578 | AB595137 | 1501 |
| *Aggregatibacter actinomycetemcomitans* NUM4039 | AB512007 | 1508 |
| *Aggregatibacter actinomycetemcomitans* NUM-Aa5014 | AB579013 | 1533 |
| *Aggregatibacter actinomycetemcomitans* OMZ534 | AB512009 | 1534 |
| *Campylobacter avium* 86/06 | EU623473 | 1354 |

| | | |
|---|---|---|
| *Campylobacter canadensis* L266 | EF621894 | 1361 |
| *Campylobacter coli* LMG6440 | AF372092 | 1341 |
| *Campylobacter concisus* ATCC33237 | DQ174166 | 1340 |
| *Campylobacter cuniculorum* 150B | DQ400345 | 1390 |
| *Campylobacter curvus* ATCC35224 | DQ174165 | 1479 |
| *Campylobacter fetus* ATCC27374 | DQ174127 | 1343 |
| *Campylobacter fetus* NCTC10354 | DQ174131 | 1343 |
| *Campylobacter gracilis* ATCC33236 | DQ174168 | 1341 |
| *Campylobacter helveticus* NCTC12470 | U03022 | 1439 |
| Campylobacter hominis NCTC13146 | AJ251584 | 1414 |
| *Campylobacter hyointestinalis* CHY5 | AF097685 | 1419 |
| *Campylobacter hyointestinalis* NCTC11608 | AF097689 | 1423 |
| *Campylobacter insulaenigrae* NCTC12927 | DQ174183 | 1341 |
| *Campylobacter jejuni* LMG8843 | DQ174144 | 1341 |
| *Campylobacter jejuni* NCTC11351 | AF372091 | 1341 |
| *Campylobacter lanienae* NCTC13004 | AF043425 | 1467 |
| *Campylobacter lari* ATCC35221 | AY621114 | 1441 |
| *Campylobacter lari* LMG21009 | AM922330 | 1501 |
| *Campylobacter mucosalis* ATCC43264 | DQ174173 | 1339 |
| *Campylobacter peloridis* LMG23910 | AM922331 | 1497 |
| *Campylobacter rectus* CCUG20446B | DQ174169 | 1339 |
| *Campylobacter showae* CCUG30254 | DQ174155 | 1339 |
| *Campylobacter sputorum biovar sputorum* LMG7795 | X67775 | 1695 |
| *Campylobacter sputorum* NCTC11367 | DQ174151 | 1570 |
| *Campylobacter subantarcticus* LMG24377 | AM933371 | 1485 |
| *Campylobacter upsaliensis* CCUG14913 | DQ174157 | 1341 |
| *Campylobacter ureolyticus* ATCC33387 | L04321 | 1453 |
| *Campylobacter volucris* LMG24380 | FM883694 | 1483 |
| *Capnocytophaga canimorsus* ATCC35979 | X97246 | 1423 |
| *Capnocytophaga cynodegmi* ATCC49044 | X97245 | 1427 |

| | | |
|---|---|---|
| *Capnocytophaga genomosp. C1* C2MKM106 | AY278613 | 1440 |
| *Capnocytophaga gingivalis* ATCC33624 | X67608 | 1453 |
| *Capnocytophaga granulosa* ATCC51502 | X97248 | 1407 |
| *Capnocytophaga haemolytica* ATCC51501 | X97247 | 1379 |
| *Capnocytophaga leadbetteri* AHN8855 | DQ009623 | 1487 |
| *Capnocytophaga ochracea* ATCC33596 | X67610 | 1489 |
| *Capnocytophaga sputigena* ATCC33612 | X67609 | 1489 |
| *Eikenella corrodens* 1664276 | AF320620 | 1476 |
| *Eikenella corrodens* ATCC23834 | M22512 | 1481 |
| *Eikenella corrodens* FDC1073 | M22515 | 1476 |
| *Eikenella corrodens* GHG7 | GU561426 | 1433 |
| *Eikenella corrodens* JCM12952 | AB525415 | 1503 |
| *Eikenella corrodens* MooreD25 | GQ422740 | 1516 |
| *Eikenella corrodens* OI061 | JN713283 | 1534 |
| *Eikenella* sp. | L06165 | 1489 |
| *Eikenella sp. canine oral taxon 049* OB066 | JN713218 | 1532 |
| *Eikenella* sp. MDA2346-4 | AY286546 | 1492 |
| *Fusobacterium canifelinum* RMA1036 | AY162221 | 1458 |
| *Fusobacterium equinum* VPB4027 | AJ295750 | 1472 |
| *Fusobacterium gonidiaformans* ATCC25563T | X55410 | 1333 |
| *Fusobacterium mortiferum* ATCC25557 | AJ867032 | 1495 |
| *Fusobacterium necrogenes* ATCC25556 | AJ867034 | 1456 |
| *Fusobacterium necrophorum* ATCC25286 | AJ867039 | 1455 |
| *Fusobacterium necrophorum* DSM19678 | AM905356 | 1487 |
| *Fusobacterium nucleatum* ATCC10953 | AF287812 | 1442 |
| *Fusobacterium nucleatum* ATCC25586 | AE009951 | 1520 |
| *Fusobacterium nucleatum* NCTC11326T | X55403 | 1331 |
| *Fusobacterium nucleatum* NCTC12276T | X55404 | 1331 |
| *Fusobacterium nucleatum subsp. animalis* RMA6247 | HM347076 | 1396 |
| *Fusobacterium nucleatum subsp. fusiforme* ATCC51190 | HM347073 | 1400 |

| | | |
|---|---|---|
| *Fusobacterium nucleatum subsp. nucleatum* ATCC25586 | GQ301038 | 1442 |
| *Fusobacterium nucleatum subsp. nucleatum* ATCC25586T | X55401 | 1331 |
| *Fusobacterium nucleatum subsp. polymorphum* ATCC10953 | GQ301039 | 1428 |
| *Fusobacterium nucleatum subsp. polymorphum* WAL10113 | HM347056 | 1372 |
| *Fusobacterium nucleatum subsp. polymorphum* WAL10141 | HM347055 | 1357 |
| *Fusobacterium nucleatum subsp. vincentii* ATCC49256 | GQ301040 | 1415 |
| *Fusobacterium nucleatum* YWH7388 | KF444254 | 1394 |
| *Fusobacterium perfoetens* ATCC29250 | M58684 | 1458 |
| *Fusobacterium periodonticum* ATCC33693T | X55405 | 1331 |
| *Fusobacterium russii* ATCC25533 | M58681 | 1453 |
| *Fusobacterium simiae* ATCC33568 | M58685 | 1468 |
| *Fusobacterium ulcerans* NCTC12111T | X55412 | 1333 |
| *Fusobacterium varium* ATCC8501 | AJ867036 | 1493 |
| *Gemella asaccharolytica* WAL1945J | EU427463 | 1404 |
| *Gemella bergeri* 617-93 | Y13365 | 1508 |
| *Gemella cuniculi* M60449/99/1 T | AJ251987 | 1451 |
| *Gemella haemolysans* ATCC10379 | L14326 | 1537 |
| *Gemella morbillorum* | L14327 | 1536 |
| *Gemella palaticanis* M663-98-1 | Y17280 | 1375 |
| *Gemella sanguinis* 2045-94 | Y13364 | 1510 |
| *Haemophilus parainfluenzae* 212335 | AY365452 | 1275 |
| *Haemophilus parainfluenzae* 225322 | AY365450 | 1322 |
| *Haemophilus parainfluenzae* ATCC33392 | M75081 | 1483 |
| *Haemophilus parainfluenzae* B160041 | FJ685628 | 1531 |
| *Haemophilus parainfluenzae* CCUG12836 | AY362908 | 1362 |
| *Haemophilus parainfluenzae* CIP102513 | EU083530 | 1505 |
| *Haemophilus parainfluenzae* GRG10 | GU561422 | 1420 |
| *Haemophilus parainfluenzae* HK102 | JF506650 | 1362 |
| *Haemophilus parainfluenzae* NCTC10672 | JF506651 | 1363 |
| *Haemophilus parainfluenzae* PN116 | FJ939586 | 1362 |

| | | |
|---|---|---|
| *Lactobacillus acidophilus* | M58802 | 1569 |
| *Lactobacillus acidophilus* ATCC4356 | AB008203 | 1553 |
| *Lactobacillus acidophilus* BCRC10695 | AY773947 | 1478 |
| *Lactobacillus acidophilus* BMF6Lb6 | M99704 | 1568 |
| *Lactobacillus acidophilus* CH-2 | EU483105 | 1478 |
| *Lactobacillus acidophilus* IDCC3301 | EF533992 | 1521 |
| *Lactobacillus acidophilus* KLDS1.0732 | EU626017 | 1469 |
| *Lactobacillus acidophilus* LH4 | AY763429 | 1462 |
| *Lactobacillus acidophilus* NCDO1748 | X61138 | 1379 |
| *Lactobacillus acidophilus* NX2-6 | EU878007 | 1481 |
| *Leptotrichia buccalis* | L37788 | 1468 |
| *Leptotrichia buccalis* DSM1135 | CP001685 | 1512 |
| *Leptotrichia buccalis* GEJ9 | GU561361 | 1396 |
| *Leptotrichia buccalis* HKU27 | GU086178 | 1311 |
| *Leptotrichia buccalis* JCM12969 | AB818949 | 1473 |
| *Leptotrichia buccalis* NCTC10429 | X90831 | 1327 |
| *Leptotrichia goodfellowii* LB57 | AY029807 | 1485 |
| *Leptotrichia hofstadii* LB23 | AY029803 | 1486 |
| *Leptotrichia hongkongensis* HKU24 | EU919515 | 1268 |
| *Leptotrichia shahii* LB37 | AY029806 | 1485 |
| *Leptotrichia sp. oral isolate* A39FD | AF287816 | 1449 |
| *Leptotrichia sp. oral strain* FAC5 | AF287813 | 1447 |
| *Leptotrichia sp. oral taxon 223* F138 | GU408547 | 1432 |
| *Leptotrichia sp. oral taxon 847* F0260 | FJ577250 | 1485 |
| *Leptotrichia trevisanii* | AF206305 | 1461 |
| *Leptotrichia wadei* LB16 | AY029802 | 1486 |
| *Parvimonas micra* ATCC33270 | AY323523 | 1481 |
| *Parvimonas micra* ATCC33270 | AF542231 | 1414 |
| *Parvimonas micra* HG1259 | AY435495 | 1310 |
| *Parvimonas micra* JCM12970 | AB729072 | 1488 |

| | | |
|---|---|---|
| *Parvimonas micra* OC026 | JN713239 | 1517 |
| *Porphyromonas gingivalis* | AF287987 | 1450 |
| *Porphyromonas gingivalis* 1-PGI | AF414810 | 1363 |
| *Porphyromonas gingivalis* 40-PGI | AF414813 | 1363 |
| *Porphyromonas gingivalis* ATCC33277 | L16492 | 1474 |
| *Porphyromonas gingivalis* ATCC33277 | AP009380 | 1533 |
| *Porphyromonas gingivalis* ATCC33277 | AB035459 | 1472 |
| *Porphyromonas gingivalis* ATCC33277 | AF285870 | 1462 |
| *Porphyromonas gingivalis* ATCC33277 | AF414809 | 1363 |
| *Porphyromonas gingivalis* DSM20709 | X73964 | 1453 |
| *Porphyromonas gingivalis* FDC381 | AB035455 | 1472 |
| *Porphyromonas gingivalis* JCM12257 | AB547660 | 1493 |
| *Porphyromonas gingivalis* JCM8525 | AB547661 | 1481 |
| *Porphyromonas gingivalis* SUNY1021 | AB035457 | 1472 |
| *Porphyromonas gingivalis* TDC60 | AP012203 | 1532 |
| *Porphyromonas gingivalis* W83 | AB035456 | 1472 |
| *Porphyromonas gingivalis* W83 | AE015924 | 1532 |
| *Prevotella denticola* ATCC33185 | L16466 | 1479 |
| *Prevotella denticola* ATCC35308 | L16467 | 1474 |
| *Prevotella denticola* ATCC35308 | AY323524 | 1506 |
| *Prevotella denticola* ChDCB698 | KF933799 | 1492 |
| *Prevotella denticola* F0289 | GU470898 | 1505 |
| *Prevotella denticola* F0289 | CP002589 | 1532 |
| *Prevotella denticola* JCM13449 | AB547679 | 1492 |
| *Prevotella denticola* JCM8528 | AB547680 | 1479 |
| *Prevotella denticola* SEQ063 | JN867232 | 1456 |
| *Prevotella denticola* SEQ076 | JN867242 | 1225 |
| *Prevotella denticola* SEQ188 | JN867263 | 1434 |
| *Prevotella denticola* SEQ197 | JN867272 | 1439 |
| *Prevotella denticola* SEQ202 | JN867277 | 1439 |

| | | |
|---|---|---|
| *Prevotella denticola* SEQ210 | JN867285 | 1440 |
| *Prevotella denticola* SEQ247 | JN867320 | 1433 |
| *Prevotella denticola* ST27 | GU561351 | 1255 |
| *Prevotella intermedia* 10-PIN | AF414827 | 1364 |
| *Prevotella intermedia* 15-PIN | AF414828 | 1364 |
| *Prevotella intermedia* 16-PIN | AF414829 | 1364 |
| *Prevotella intermedia* 22-PIN | AF414830 | 1364 |
| *Prevotella intermedia* 23-PIN | AF414831 | 1364 |
| *Prevotella intermedia* 24-PIN | AF414832 | 1364 |
| *Prevotella intermedia* 32-PIN | AF414824 | 1364 |
| *Prevotella intermedia* 35-PIN | AF414825 | 1364 |
| *Prevotella intermedia* 36-PIN | AF414826 | 1364 |
| *Prevotella intermedia* 5-PIN | AF414822 | 1364 |
| *Prevotella intermedia* 6-PIN | AF414823 | 1364 |
| *Prevotella intermedia* ATCC25611 | L16468 | 1473 |
| *Prevotella intermedia* ATCC25611 | X73965 | 1459 |
| *Prevotella intermedia* ATCC25611 | AF414821 | 1364 |
| *Prevotella intermedia* ChDC KB14 | AY689222 | 1495 |
| *Prevotella intermedia* ChDC KB18 | AY689223 | 1494 |
| *Prevotella intermedia* ChDC KB2 | AY689220 | 1494 |
| *Prevotella intermedia* ChDC KB29 | AY689225 | 1494 |
| *Prevotella intermedia* ChDC KB3 | AY689221 | 1494 |
| *Prevotella intermedia* ChDC KB53 | AY689226 | 1495 |
| *Prevotella melaninogenica* ATCC25845 | CP002122 | 1531 |
| *Prevotella melaninogenica* ATCC25845 | L16469 | 1478 |
| *Prevotella melaninogenica* ATCC25845 | CP002123 | 1532 |
| *Prevotella melaninogenica* ATCC25845 | AY323525 | 1506 |
| *Prevotella melaninogenica* ATCC43982 | L16470 | 1473 |
| *Prevotella melaninogenica* ATCC43982 | FJ792538 | 1372 |
| *Prevotella melaninogenica* C561 | JF803512 | 1479 |

| | | |
|---|---|---|
| *Prevotella melaninogenica* H2_18 | GU561346 | 1411 |
| *Prevotella melaninogenica* JCM6321 | AB547694 | 1479 |
| *Prevotella melaninogenica* JCM6324 | AB686661 | 1492 |
| *Prevotella melaninogenica* JCM6325 | AB547693 | 1480 |
| *Prevotella melaninogenica* SEQ074 | JN867240 | 1436 |
| *Prevotella melaninogenica* SEQ115 | JN867245 | 1366 |
| *Prevotella melaninogenica* SEQ117 | JN867247 | 1440 |
| *Prevotella melaninogenica* SEQ182 | JN867258 | 1326 |
| *Prevotella melaninogenica* SEQ189 | JN867264 | 1424 |
| *Prevotella melaninogenica* SEQ220 | JN867295 | 1432 |
| *Prevotella melaninogenica* SEQ236 | JN867309 | 1431 |
| *Prevotella melaninogenica* SEQ244 | JN867317 | 1430 |
| *Prevotella melaninogenica* SG14 | GU561347 | 1280 |
| *Prevotella melaninogenica*DNF00666 | KF280298 | 1286 |
| *Propionibacterium acidifaciens* C3M_31 | EU979537 | 1443 |
| *Propionibacterium acidipropionici* NCFB570 | AJ704570 | 1440 |
| *Propionibacterium acnes* ATCC6919 | AB042288 | 1486 |
| *Propionibacterium australiense* LCDC-98A072 | AF225962 | 1469 |
| *Propionibacterium avidum* DSM4901 | AJ003055 | 1504 |
| *Propionibacterium cyclohexanicum* | D82046 | 1471 |
| *Propionibacterium freudenreichii* DSM20271 | X53217 | 1462 |
| *Propionibacterium freudenreichii* DSM4902 | Y10819 | 1510 |
| *Propionibacterium granulosum* DSM20700 | AJ003057 | 1514 |
| *Propionibacterium jensenii* DSM20535 | AJ704571 | 1487 |
| *Propionibacterium microaerophilum* M5 | AF234623 | 1524 |
| *Propionibacterium propionicum* DSM43307 | AJ003058 | 1506 |
| *Propionibacterium thoenii* NCFB568 | AJ704572 | 1467 |
| *Selenomonas artemidis* ATCC43528 | GQ422716 | 1521 |
| *Selenomonas bovis* WG | EF139191 | 1525 |
| *Selenomonas dianae* ATCC43527 | AF287801 | 1494 |

| | | |
|---|---|---|
| *Selenomonas flueggei* ATCC43531 | AF287803 | 1488 |
| *Selenomonas infelix* ATCC43532 | AF287802 | 1524 |
| *Selenomonas noxia* ATCC43541 | AF287799 | 1491 |
| *Selenomonas ruminantium* GA192 | M62702 | 1486 |
| *Selenomonas ruminantium* JCM6582 | AB003379 | 1455 |
| *Selenomonas sputigena* ATCC35185 | AF287793 | 1497 |
| *Staphylococcus arlettae* ATCC43957 | AB009933 | 1494 |
| *Staphylococcus aureus* ATCC12600 | L36472 | 1555 |
| *Staphylococcus aureus* 007.5 | EU515208 | 1479 |
| *Staphylococcus aureus* ATCC14458 | DQ997837 | 1516 |
| *Staphylococcus aureus* ATCC29740 | AF015929 | 1442 |
| *Staphylococcus aureus* ATCC35844 | D83355 | 1476 |
| Staphylococcus aureus ATCC43300 | AM980864 | 1519 |
| *Staphylococcus aureus* EHFS1_S02Ha | EU071602 | 1416 |
| *Staphylococcus aureus* F41 | DQ997832 | 1473 |
| *Staphylococcus aureus* MPU99 | AB353073 | 1500 |
| *Staphylococcus aureus* OA1 | D83356 | 1476 |
| *Staphylococcus aureus* RKA4 | EF463058 | 1415 |
| *Staphylococcus aureus* S2 | FJ434467 | 1417 |
| *Staphylococcus aureus* SGC801 | GQ919290 | 1461 |
| *Staphylococcus aureus* SMKV-1 | DQ306890 | 1507 |
| *Staphylococcus auricularis* ATCC33753 | D83358 | 1475 |
| *Staphylococcus capitis* | L37599 | 1469 |
| *Staphylococcus capitis* ATCC49326 | AB009937 | 1492 |
| *Staphylococcus caprae* ATCC35538 | AB009935 | 1492 |
| *Staphylococcus carnosus* GTC1232 | AB233329 | 1454 |
| *Staphylococcus carnosus* ATCC51365 | AB009934 | 1493 |
| *Staphylococcus chromogenes* ATCC43764 | D83360 | 1475 |
| *Staphylococcus cohnii* ATCC29974 | D83361 | 1477 |
| *Staphylococcus cohnii* ATCC49330 | AB009936 | 1494 |

| | | |
|---|---|---|
| *Staphylococcus condimenti* DSM11674T | Y15750 | 1545 |
| *Staphylococcus delphini* ATCC49171 | AB009938 | 1493 |
| *Staphylococcus devriesei* KS-SP_60 | FJ389206 | 1537 |
| *Staphylococcus epidermidis* ATCC14990 | D83363 | 1475 |
| *Staphylococcus equorum* ATCC43958 | AB009939 | 1494 |
| *Staphylococcus equorum* RP29 | AF527483 | 1535 |
| *Staphylococcus felis* ATCC 49168 | D83364 | 1475 |
| *Staphylococcus fleurettii* GTC1999 | AB233330 | 1454 |
| *Staphylococcus gallinarum* ATCC35539 | D83366 | 1477 |
| *Staphylococcus haemolyticus* CCM2737 | X66100 | 1544 |
| *Staphylococcus hominis* DSM20328 | X66101 | 1544 |
| *Staphylococcus hominis* GTC1228 | AB233326 | 1454 |
| *Staphylococcus hyicus* ATCC11249 | D83368 | 1476 |
| *Staphylococcus intermedius* ATCC29663 | D83369 | 1476 |
| *Staphylococcus kloosii* ATCC43959 | AB009940 | 1494 |
| *Staphylococcus lentus* ATCC29070 | D83370 | 1480 |
| *Staphylococcus lugdunensis* ATCC43809 | AB009941 | 1492 |
| *Staphylococcus lutrae* | X84731 | 1511 |
| *Staphylococcus massiliensis* CCUG55927 | EU707796 | 1477 |
| *Staphylococcus microti* CCM4903 | EU888120 | 1477 |
| *Staphylococcus nepalensis* CW1 | AJ517414 | 1470 |
| *Staphylococcus pasteuri* ATCC51129 | AB009944 | 1494 |
| *Staphylococcus pettenkoferi* B3117 | AF322002 | 1547 |
| *Staphylococcus piscifermentans* SK03 T | Y15754 | 1544 |
| *Staphylococcus pseudintermedius* LMG22219 | AJ780976 | 1512 |
| *Staphylococcus rostri* ARI262 | FM242137 | 1413 |
| *Staphylococcus saccharolyticus* | L37602 | 1527 |
| *Staphylococcus saprophyticus* GTC843 | AB233327 | 1454 |
| *Staphylococcus saprophyticus* ATCC15305 | AP008934 | 1555 |
| *Staphylococcus schleiferi* | S83568 | 1554 |

| | | |
|---|---|---|
| *Staphylococcus schleiferi* ATCC49545 | AB009945 | 1492 |
| *Staphylococcus sciuri* DSM20345T | AJ421446 | 1528 |
| *Staphylococcus sciuri* GTC1227 | AB233331 | 1454 |
| *Staphylococcus sciuri* GTC844 | AB233332 | 1454 |
| *Staphylococcus simiae* CCM7213 | AY727530 | 1478 |
| *Staphylococcus simulans* ATCC27848 | D83373 | 1476 |
| *Staphylococcus succinus* SB72 | AJ320272 | 1535 |
| *Staphylococcus succinus* AMG-D1 | AF004220 | 1548 |
| *Staphylococcus vitulinus* ATCC51145 | AB009946 | 1497 |
| *Staphylococcus warneri* | L37603 | 1470 |
| *Staphylococcus xylosus* ATCC29971 | D83374 | 1477 |
| *Streptococcus equinus* ATCC9812 | AJ301607 | 1433 |
| *Streptococcus henryi* 126 | EF364097 | 1430 |
| *Streptococcus infantarius* HDP90246 | AF429763 | 1473 |
| *Streptococcus infantarius* HDP90104 | AF177729 | 1493 |
| *Streptococcus infantis* ATCC700779 | AY485603 | 1468 |
| *Streptococcus lactarius* MV1 | GU045364 | 1453 |
| *Streptococcus lutetiensis* NEM782 | AJ297215 | 1461 |
| *Streptococcus macacae* ATCC35911 | AY188351 | 1547 |
| *Streptococcus mitis* ATCC49456 | AF003929 | 1520 |
| *Streptococcus mitis* ATCC15914 | AY281076 | 1430 |
| *Streptococcus mitis* ATCC49456 | AY485601 | 1469 |
| *Streptococcus mitis* ATCC6249 | AY281077 | 1428 |
| *Streptococcus mitis* ATCC903 | AY281078 | 1430 |
| *Streptococcus mutans* 669 | AF139603 | 1551 |
| *Streptococcus mutans* AF199 | AF139602 | 1551 |
| *Streptococcus mutans* LM7 | AF139601 | 1551 |
| *Streptococcus mutans* NCTC10449 | AJ243965 | 1512 |
| *Streptococcus mutans* UA140 | AF139599 | 1551 |
| *Streptococcus oralis* ATCC35037 | AY485602 | 1471 |

| | | |
|---|---|---|
| *Streptococcus oralis* ATCC35037 | AF003932 | 1460 |
| *Streptococcus oralis* ATCC700233 | AY281080 | 1428 |
| Streptococcus oralis ATCC9811 | AY281079 | 1428 |
| *Streptococcus oralis* CCUG24891 | DQ303190 | 1473 |
| *Streptococcus peroris* GTC848 | AB008314 | 1339 |
| *Streptococcus pneumoniae* ATCC33400 | AF003930 | 1515 |
| *Streptococcus pseudopneumoniae* ATCC BAA-960 | AY612844 | 1468 |
| *Streptococcus ursoris* NUM1615 | AB501126 | 1550 |
| *Treponema denticola* ATCC35405 | AE017226 | 1546 |
| *Treponema denticola* ATCC33520 | D85438 | 1376 |
| *Treponema denticola* ATCC35404 | AF139204 | 1450 |
| *Treponema denticola* ATCC35405 | AF139203 | 1518 |
| *Treponema denticola* C057 | GU416568 | 1457 |
| *Treponema denticola* D011 | GU416610 | 1458 |
| *Treponema denticola* GF012 | GU416600 | 1479 |
| *Treponema denticola* GM-1 | AY119692 | 1365 |
| *Treponema denticola* PY004 | JN713361 | 1496 |
| *Treponema denticola* T009 | GU416613 | 1453 |
| *Treponema denticola* U061A | GU416572 | 1459 |
| *Veillonella atypica* ATCC17744 | AF439641 | 1400 |
| *Veillonella caviae* DSM20738 | AY355140 | 1402 |
| *Veillonella criceti* ATCC17747 | AF186072 | 1492 |
| *Veillonella denticariosi* RBV106 | EF185167 | 1497 |
| *Veillonella dispar* ATCC17748 | AF439639 | 1375 |
| *Veillonella magna* lac18 | EU096495 | 1449 |
| *Veillonella montpellierensis* ADV281.99 | AF473836 | 1409 |
| *Veillonella parvula* ATCC10790 | AY995767 | 1497 |
| *Veillonella ratti* DSM20736 | AY355138 | 1396 |
| *Veillonella rodentium* ATCC17743 | AY514996 | 1307 |
| *Veillonella rogosae* CF100 | EF108443 | 1344 |

**Supplementary Figure S1: Cluster analysis of OralArray reproducibility on oral clinical samples.** (a) Cluster analysis of the samples reported in Figure 3 and stability values as calculated by pvclust. Red values ("AU" values) were used to determine cluster stability; (b) pvclust-computed standard error associated to cluster stability measures; (c) Full table of pvclust bootstrapping analysis on the samples. "AU" (approximately unbiased) is computed by multiscale bootstrap resampling of data, whereas "BP" (bootstrap probability) is computed by normal bootstrap resampling. AU values are generally considered as a better approximation to unbiased stability than BP. For a full description of table columns, please refer to pvclust documentation (https://cran.r-project.org/web/packages/pvclust/pvclust.pdf).

a

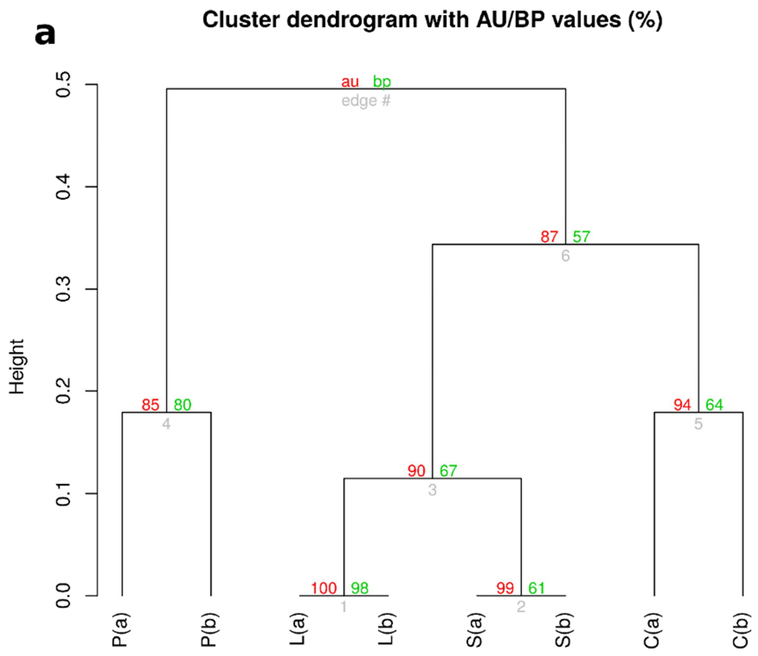


b

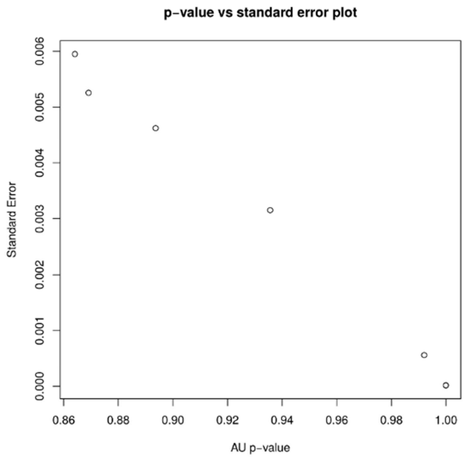


c

| Cluster node | au | bp | se.au | se.bp | v | c | pchi |
|---|---|---|---|---|---|---|---|
| 1 | 1 | 0.984 | 0 | 0.001 | -2.999 | 0.851 | 0.668 |
| 2 | 0.992 | 0.611 | 0.001 | 0.002 | -1.346 | 1.063 | 0.107 |
| 3 | 0.894 | 0.665 | 0.005 | 0.002 | -0.837 | 0.41 | 0.01 |
| 4 | 0.864 | 0.8 | 0.006 | 0.001 | -0.971 | 0.128 | 0.109 |
| 5 | 0.936 | 0.638 | 0.003 | 0.002 | -0.937 | 0.582 | 0.012 |
| 6 | 0.869 | 0.569 | 0.005 | 0.002 | -0.648 | 0.475 | 0.032 |
| 7 | 1 | 0.996 | 0 | 0 | -3.271 | 0.613 | 0.613 |

**Supplementary Figure S2: Schematic representation of the Universal Array (UA) layout.** Each slide contains 8 UAs, each constituted by 208 spots, arranged in a 13 × 16 matrix. On the right, a magnification of the location of Zip Codes and controls is reported, together with the nucleotidic sequence of the Zip Codes

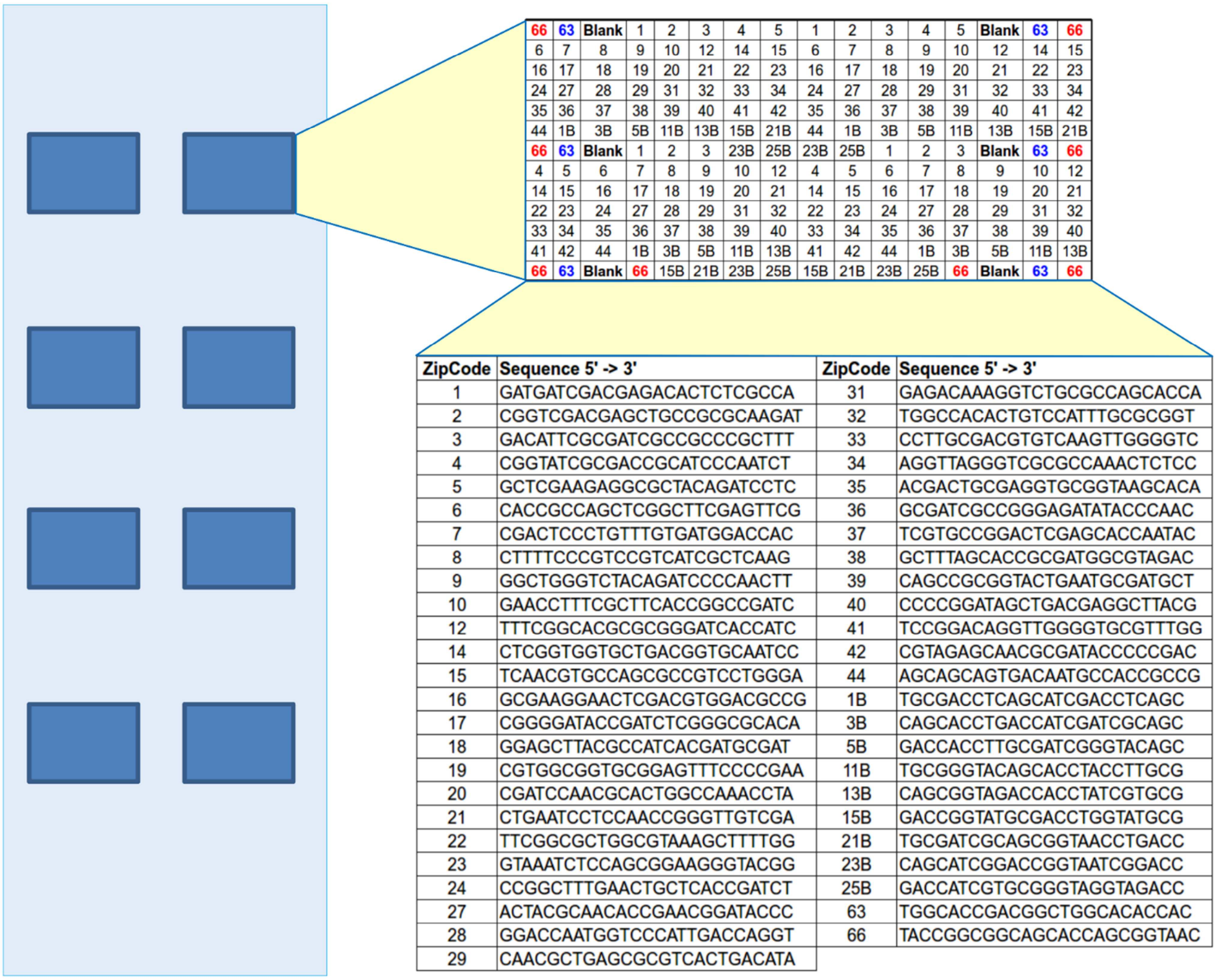

| | | | | | | | | | | | | | | | |
|---|---|---|---|---|---|---|---|---|---|---|---|---|---|---|---|
| 66 | 63 | Blank | 1 | 2 | 3 | 4 | 5 | 1 | 2 | 3 | 4 | 5 | Blank | 63 | 66 |
| 6 | 7 | 8 | 9 | 10 | 12 | 14 | 15 | 6 | 7 | 8 | 9 | 10 | 12 | 14 | 15 |
| 16 | 17 | 18 | 19 | 20 | 21 | 22 | 23 | 16 | 17 | 18 | 19 | 20 | 21 | 22 | 23 |
| 24 | 27 | 28 | 29 | 31 | 32 | 33 | 34 | 24 | 27 | 28 | 29 | 31 | 32 | 33 | 34 |
| 35 | 36 | 37 | 38 | 39 | 40 | 41 | 42 | 35 | 36 | 37 | 38 | 39 | 40 | 41 | 42 |
| 44 | 1B | 3B | 5B | 11B | 13B | 15B | 21B | 44 | 1B | 3B | 5B | 11B | 13B | 15B | 21B |
| 66 | 63 | Blank | 1 | 2 | 3 | 23B | 25B | 23B | 25B | 1 | 2 | 3 | Blank | 63 | 66 |
| 4 | 5 | 6 | 7 | 8 | 9 | 10 | 12 | 4 | 5 | 6 | 7 | 8 | 9 | 10 | 12 |
| 14 | 15 | 16 | 17 | 18 | 19 | 20 | 21 | 14 | 15 | 16 | 17 | 18 | 19 | 20 | 21 |
| 22 | 23 | 24 | 27 | 28 | 29 | 31 | 32 | 22 | 23 | 24 | 27 | 28 | 29 | 31 | 32 |
| 33 | 34 | 35 | 36 | 37 | 38 | 39 | 40 | 33 | 34 | 35 | 36 | 37 | 38 | 39 | 40 |
| 41 | 42 | 44 | 1B | 3B | 5B | 11B | 13B | 41 | 42 | 44 | 1B | 3B | 5B | 11B | 13B |
| 66 | 63 | Blank | 66 | 15B | 21B | 23B | 25B | 15B | 21B | 23B | 25B | 66 | Blank | 63 | 66 |

| ZipCode | Sequence 5' -> 3' | ZipCode | Sequence 5' -> 3' |
|---|---|---|---|
| 1 | GATGATCGACGAGACACTCTCGCCA | 31 | GAGACAAAGGTCTGCGCCAGCACCA |
| 2 | CGGTCGACGAGCTGCCGCGCAAGAT | 32 | TGGCCACACTGTCCATTTGCGCGGT |
| 3 | GACATTCGCGATCGCCGCCCGCTTT | 33 | CCTTGCGACGTGTCAAGTTGGGGTC |
| 4 | CGGTATCGCGACCGCATCCCAATCT | 34 | AGGTTAGGGTCGCGCCAAACTCTCC |
| 5 | GCTCGAAGAGGCGCTACAGATCCTC | 35 | ACGACTGCGAGGTGCGGTAAGCACA |
| 6 | CACCGCCAGCTCGGCTTCGAGTTCG | 36 | GCGATCGCCGGGAGATATACCCAAC |
| 7 | CGACTCCCTGTTTGTGATGGACCAC | 37 | TCGTGCCGGACTCGAGCACCAATAC |
| 8 | CTTTTCCCGTCCGTCATCGCTCAAG | 38 | GCTTTAGCACCGCGATGGCGTAGAC |
| 9 | GGCTGGGTCTACAGATCCCCAACTT | 39 | CAGCCGCGGTACTGAATGCGATGCT |
| 10 | GAACCTTTCGCTTCACCGGCCGATC | 40 | CCCCGGATAGCTGACGAGGCTTACG |
| 12 | TTTCGGCACGCGCGGGATCACCATC | 41 | TCCGGACAGGTTGGGGTGCGTTTGG |
| 14 | CTCGGTGGTGCTGACGGTGCAATCC | 42 | CGTAGAGCAACGCGATACCCCCGAC |
| 15 | TCAACGTGCCAGCGCCGTCCTGGGA | 44 | AGCAGCAGTGACAATGCCACCGCCG |
| 16 | GCGAAGGAACTCGACGTGGACGCCG | 1B | TGCGACCTCAGCATCGACCTCAGC |
| 17 | CGGGGATACCGATCTCGGGCGCACA | 3B | CAGCACCTGACCATCGATCGCAGC |
| 18 | GGAGCTTACGCCATCACGATGCGAT | 5B | GACCACCTTGCGATCGGGTACAGC |
| 19 | CGTGGCGGTGCGGAGTTTCCCCGAA | 11B | TGCGGGTACAGCACCTACCTTGCG |
| 20 | CGATCCAACGCACTGGCCAAACCTA | 13B | CAGCGGTAGACCACCTATCGTGCG |
| 21 | CTGAATCCTCCAACCGGGTTGTCGA | 15B | GACCGGTATGCGACCTGGTATGCG |
| 22 | TTCGGCGCTGGCGTAAAGCTTTTGG | 21B | TGCGATCGCAGCGGTAACCTGACC |
| 23 | GTAAATCTCCAGCGGAAGGGTACGG | 23B | CAGCATCGGACCGGTAATCGGACC |
| 24 | CCGGCTTTGAACTGCTCACCGATCT | 25B | GACCATCGTGCGGGTAGGTAGACC |
| 27 | ACTACGCAACACCGAACGGATACCC | 63 | TGGCACCGACGGCTGGCACACCAC |
| 28 | GGACCAATGGTCCCATTGACCAGGT | 66 | TACCGGCGGCAGCACCAGCGGTAAC |
| 29 | CAACGCTGAGCGCGTCACTGACATA | | |
